# Multipoint Anionic Bridge: Asymmetric Solvation Structure Improves the Stability of Lithium‐Ion Batteries

**DOI:** 10.1002/advs.202410329

**Published:** 2024-10-30

**Authors:** Tianle Zheng, Tonghui Xu, Jianwei Xiong, Weiping Xie, Mengqi Wu, Ying Yu, Zhuijun Xu, Yuxin Liang, Can Liao, Xiaoli Dong, Yongyao Xia, Ya‐Jun Cheng, Yonggao Xia, Peter Müller‐Buschbaum

**Affiliations:** ^1^ Chair for Functional Materials Department of Physics TUM School of Natural Sciences Technical University of Munich James‐Franck‐Str. 1 85748 Garching Germany; ^2^ Ningbo Institute of Materials Technology and Engineering Chinese Academy of Sciences 1219 Zhongguan West Rd Ningbo Zhejiang 315201 P. R. China; ^3^ Department of Chemistry College of Sciences Shanghai University Shanghai 200444 P. R. China; ^4^ College of Materials Science and Technology Nanjing University of Aeronautics and Astronautics Nanjing 210016 P. R. China; ^5^ AIE Institute Guangdong 510530 P. R. China; ^6^ College of Materials Science and Engineering Fuzhou University Fuzhou Fujian 350108 P. R. China; ^7^ Department of Chemistry Institute of New Energy Fudan University Shanghai 200433 P. R. China; ^8^ College of Renewable Energy Hohai University Jiangsu 213220 P. R. China; ^9^ Center of Materials Science and Optoelectronics Engineering University of Chinese Academy of Sciences Beijing 100049 P. R. China

**Keywords:** asymmetric solvation structure, high temperature, high‐concentration electrolyte, lithium difluoro(oxalate)borate, lithium metal batteries

## Abstract

In this study, a novel concept of multipoint anionic bridge (MAB) is proposed and proved, which utilizes anions with different sites to connect with the asymmetric solvation structure (ASS). Compared to usual solvation structures, this study utilizes the multifunctional groups of difluoro(oxalate)borate anion (ODFB^−^), which can connect with Li^+^. By tailoring the concentration, the anion serves as a bridge between different solvated structures. The electrolyte is investigated through in situ techniques and simulations to draw correlations between different solvation structures and reaction pathways. The proposed design demonstrates remarkable high‐temperature performance on both the anode and cathode sides, enabling stable cycling of LCO||graphite (0.5 Ah, 1.0 C) pouch cell for over 200 cycles at 80 °C and facilitating Li||MCMB and Li||LFP cells to deliver stable performance for 200 cycles at 100 °C. This work paves the way for the development of high‐performance electrolyte systems by designing and using new multipoint anions to construct ASSs.

## Graphical abstract

The multipoint anionic bridge concept is proposed, which utilizes anions with multiple complex sites to establish connections within the asymmetric solvation structure. Distinguished from conventional electrolytes, this study cleverly exploits the structural characteristics of the difluoro(oxalate)borate anion, using anions as bridges to coordinate different solvation structures and achieve significant performance enhancement on both the anode and cathode sides.

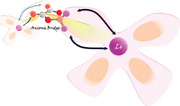

## 1 Introduction

The electrolyte, often referred to as the lifeblood of the battery, plays a crucial role in the battery system.^[^ ^1^ ^]^ Many of the current limitations electrolytes face in batteries, such as their restricted operating temperature range, limited electrochemical window, and inherent flammability, are predominantly determined by the “blood,” i.e., the electrolyte.^[^ ^2^ ^]^ Typically, electrolytes consist of lithium salts, solvents, and/or functional additives.^[^ ^3^ ^]^ In electrolytes, various interactions coexist, including cation–solvent, cation–anion, anion–solvent, and solvent–solvent interactions. The solubility of lithium salts and the cation transfer number is directly influenced by the relative strength of cation–solvent interactions compared to cation–anion complexes. Notably, the solvation structures of Li^+^ (solvent‐separated ion pairs, SSIPs; contact ion pairs, CIPs; or ion aggregates, AGGs) are determined by the relative strength of these two complexes.^[^ ^4^ ^]^

Due to the impact of solvation structure on various aspects of battery operation, such as lithium‐ion transport, electrochemical stability, and formation of solid electrolyte interface (SEI) layer, the design of the solvation shell is crucial. It has emerged as a prominent research area.^[^ ^5^ ^]^ The precise control of the solvation structure can eliminate the adverse effects of solvent molecules.^[^ ^6^ ^]^ Therefore, various alternative and novel electrolytes were reported, including high‐concentration electrolytes (HCE), polymer electrolytes (PE), and weakly solvating electrolytes (WSE), among others.^[^ ^7^ ^]^ These advancements are crucial for developing next‐generation lithium‐ion batteries. For instance, Li et al. demonstrated the indispensability of the Li‐ethylene carbonate (EC) complex in electrolytes containing EC for enhancing electrolyte stability.^[^ ^8^ ^]^ Zheng et al. harnessed the competitive evolution of solvation structure across varying temperatures to facilitate stable operation within a broad temperature range.^[^ ^9^ ^]^ HCE, WSE, and others have the potential to significantly decrease the abundance of SSIP structures and unbound solvent molecules, predominantly consisting of CIP and AGGs.^[^ ^10^ ^]^ In the majority of studies, AGGs typically involve more than two Li ions utilizing the same anion as a bridge to occupy coordination sites, thereby providing partial insights into their coordination behavior and facilitating the formation of a sustainable SEI layer.^[^ ^11^ ^]^

**Scheme 1:**
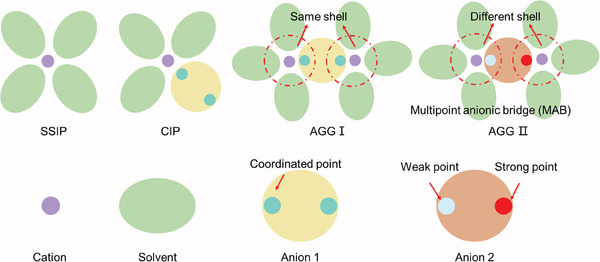
Explanation of the different solvation structures: SSIP, CIP, AGGI, and AGGII.

However, limited attention has been devoted to investigating the effect of various solvation structures of AGGs in the electrolyte. For example, Cheng et al. reported on n‐AGGs, which are significantly larger and contain tens and hundreds of ions.^[^ ^12^ ^]^ Furthermore, the presence of anion‐dominated AGGs has been observed, where the anion is selected as the central component in the solvation structure.^[^ ^13^ ^]^ However, there remains a lack of investigation into the overall evolution of solvation structures when the anion possesses weak and strong coordinate points, as shown in **Scheme** **1**, where distinct functional groups of an anion facilitate the connection between two lithium ions, resulting in the formation of two dissimilar solvated shells, asymmetric solvation structure (ASS). Thus, we chose lithium difluoro(oxalate)borate (LiODFB) as the main salt based on the hypothesis of the multipoint anionic bridge (MAB) mentioned above to connect different solvation shells. LiODFB, a hybrid salt of lithium bis(oxalate)borate (LiBOB) and lithium tetrafluoroborate (LiBF_4_), contains two functional groups, B─F and C═O, and exhibits advantageous characteristics inherited from both parent anions, such as relatively high ionic conductivity, solubility, low viscosity, and high performance.^[^ ^14^ ^]^ Moreover, the anions of lithium bis(trifluoromethanesulfonyl)imide (LiTFSI) and lithium bis (fluorosulfonyl) imide (LiFSI) exhibit multiple binding sites. However, there are significant differences in the distribution of charges between the strong and weak binding sites of FSI^−^ and TFSI^−^, which makes it virtually impossible for Li ions to interact with the weak binding sites (Figure S1, Supporting Information). Additionally, EC and propylene carbonate (PC) are commonly used solvents due to their cost‐effectiveness, high boiling points, and elevated dielectric constants, which facilitate the dissolution of LiODFB. Moreover, PC has a similar cyclic carbonate molecular structure to EC, thereby minimizing their impact on the solvated structure. Moreover, these solvents possess comparable structures and exhibit good thermal stability at elevated temperatures. In detail, different concentrations of LiODFB are dissolved in EC/PC (v/v = 1/1) cosolvent to compare the evolution of solvation structure. When the concentration is low, the solvation structure in the electrolyte exists as either SSIP or CIP. At concentrations exceeding a certain threshold, both ends of the anion functional group act as MABs to interact with lithium ions, thereby facilitating one anion to connect multiple ASSs. These ASSs will exert a substantial influence on the interface and significantly impact overall battery performance.

## 2 Result and Discussions

### 2.1 Solvation Behavior of Different Concentration Electrolytes

**Figure 1:**
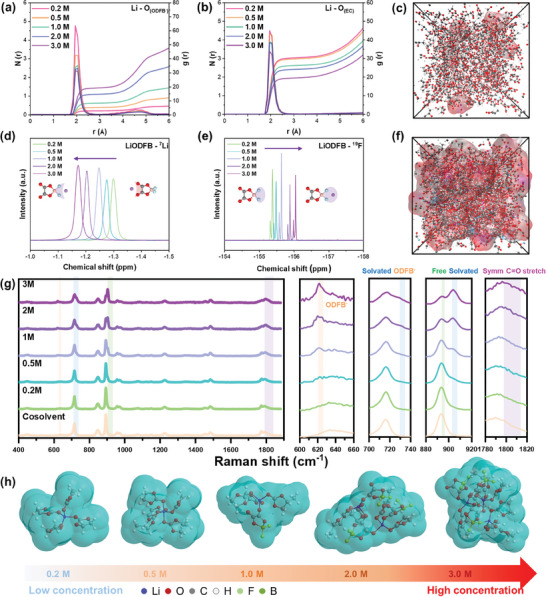
Solvation structures in electrolytes. a,b) RDF results of different concentrations of LiODFB. snapshot of c) 0.2 m and f) 3.0 m electrolytes. d) ^7^Li NMR and e)^19^F NMR of various concentrations electrolytes. g) Raman spectra of different concentrations of electrolytes and solvents. h) Evolution of solvation structures in increasing concentrations of the LiODFB electrolyte.

The solvation structure of electrolytes at different concentrations is investigated through classic molecular dynamics (MD) simulations, providing valuable insights for a comprehensive understanding. The evolution of solvation structure with increasing concentration is depicted by the radial distribution function (RDF) in **Figure** **1**a,b and Figures S2 and S3 (Supporting Information). The typical solvation structures of different electrolytes are obtained from RDFs, denoted as Li^+^(ODFB^−^)_0.2_EC_3.0_PC_1.6_, Li^+^(ODFB^−^)_0.4_EC_2.9_PC_1.5_, Li^+^(ODFB^−^)_0.6_EC_2.6_PC_1.6_, Li^+^(ODFB^−^)_1.1_EC_2.4_PC_1.4_ and Li^+^(ODFB^−^)_1.4_EC _1.9_PC_1.4_ at concentrations of 0.2, 0.5, 1.0, 2.0, and 3.0 m respectively. For Li‐O(ODFB^−^) pairs, the curve of coordination number exhibits a quasi‐linear growth trend with increasing concentration. At a concentration of 2.0 m, the value is approximately 1, while at 3.0 m, the coordination number reaches around 1.5. This indicates that when the concentration of LiODFB salt is below 2.0 m, the average solvation structure in the electrolyte fails to attain the CIP structure, whereas, in high‐concentration electrolytes exceeding 3.0 m, there exists a state where one lithium ion connects multiple anions. Conversely, the coordination number of Li–O_(EC)_ and Li–O_(PC)_ exhibited a decrease with increasing concentration, aligning with the characteristics typically observed in high‐concentration electrolytes.^[^ ^15^ ^]^ Such decline in PC was relatively smaller than that of EC, attributed to the smaller geometry hindrance of the EC molecules within the electrolyte, particularly under high viscosity conditions where this characteristic becomes more pronounced.

Furthermore, the RDF results depicted in Figure S2b,d (Supporting Information) also demonstrate that irrespective of the temperature conditions (room temperature or high temperature), the solvation structure of the electrolyte remains essentially unaltered. In summary, the impact of temperature variations on the solvation structure of the studied electrolyte system is limited. The snapshots of the dynamic trajectory of different electrolyte concentrations also reveal that as the concentration increases, the solvation structure of Li ions transitions from dispersion to aggregation, ultimately occupying nearly the entire electrolyte (Figures S4–S6, Supporting Information).

Nuclear magnetic resonance (NMR) spectroscopy is used to further substantiate the evolution of solvation structure induced by variation of the LiODFB concentration. The increase of the LiODFB concentration diminishes the number of coordinated carbonate solvent molecules to Li^+^, leading to decreased electron shielding effect around the lithium ion. With the increase in LiODFB concentrations, the local electron density around the lithium ion is not enhanced by the electrostatic interaction between the ODFB‐ and solvated lithium cation, which is attributable to the larger size of ODFB^−^. Due to these two mechanisms, a downfield chemical shift of the ^7^Li spectra with increasing LiODFB concentration is exhibited (Figure 1d), which is on the contrary to the upfield chemical shift phenomenon observed in LiPF_6_ and LiBF_4_ solutions (Figure S8, Supporting Information).^[^ ^16^ ^]^ The electron shielding effect is enhanced by the electrostatic interactions between the small sized PF_6_/BF_4_ anion and lithium cation with increasing salt concentrations. It overwhelms the decreased electron shielding effect brought by the lessened solvent molecule coordination to the lithium ion, resulting in upfield chemical shift. Furthermore, a smaller chemical shift gap of ODFB^−^ is exhibited with the concentration increased from 2.0 to 3.0 m than that of the concentration increase from 1.0 to 2.0 m. It suggests that the ODFB^−^ tends to form strong electrostatic interaction with the solvated lithium ion, leading to enhanced electron shielding compensation effect and decreased downfield chemical shift. The downfield chemical shift is also observed with the ^7^Li spectra in the LiBOB solution, which confirms the mechanism proposed for the LiODFB system because the BOB^−^ has a large size as well (Figure S8, Supporting Information). Unlike the ^7^Li spectra, an upfield chemical shift of ODFB^−^ is observed in the ^19^F spectra (Figure 1e). The electrostatic interactions among the ODFB^−^ are enhanced with increasing LiODFB concentration, resulting in a stronger electron shielding effect around the fluorine atoms. Because the two fluorine atoms within the ODFB^−^ anionic structure possess different value states (one negative and another neutral), two peaks are exhibited by the ^19^F spectra. Notably, the chemical shift gap of ^19^F decreased when the LiODFB concentration increased from 2.0 to 3.0 m compared to the chemical shift gap between 1.0 and 2.0 m. The increased LiODFB concentration induces stronger interactions between the ODFB^−^ and Li cation. The electrostatic repulsion effects among the ODFB^−^ are weakened, leading to decreased electron shielding effect and lowered chemical shift gap. This finding is consistent with the ^19^F spectra shown by the LiBF_4_ solution (Figure S9, Supporting Information). Moreover, an obvious peak widening is observed with the ^11^B spectra, which becomes even more pronounced with increasing salt concentration (Figure S10, Supporting Information). The electrostatic interactions among the ODFB^−^ become increasingly complicated as the LiODFB concentration increases. Because the boron atom is located within the large‐sized ODFB^−^, the electron shielding effect does not vary significantly with increasing LiODFB concentration. Therefore, no obvious upfield chemical shift is observed. Unlike LiODFB, the boron atom in LiBF_4_ shows a clear upfield chemical shift with increasing concentration because of its small molecular size.

Raman spectroscopy is used to investigate the evolution of solvation structure with respect to the concentration variation. As shown in Figure 1g, the gradually increasing peak located around 620 cm^−1^ is most likely attributed to the combination of B─F scissoring from the ODFB^−^ anion.^[^ ^17^ ^]^ The peak located at about 730 cm^−1^ may correspond to the C═O─Li^+^.^[^ ^17b^ ^]^ At concentrations below 1.0 m, the absence of an obvious solvent peak suggests a low number of CIP or AGG structures in the electrolyte, aligning with the findings from MD simulations. Additionally, the peak in the range of 880–920 cm^−1^ is attributed to the presence of free EC and PC molecules, while a gradually increasing solvated peak can be ascribed to the interaction between Li ions and solvent molecules, indicating that an increasing concentration of Li salts leads to enhanced solvation processes involving larger amounts of solvents.^[^ ^18^ ^]^ Moreover, the enhanced peak at 1810 cm^−1^ is ascribed to the symmetric C═O moieties of ODFB^−^.^[^ ^19^ ^]^ Symmetry is restored when Li ions complexes with each C═O group of ODFB^−^. It is worth noting that the shapes of 2.0 and 3.0 m are similar, possibly due to the saturation of coordination number of anions’ C═O with Li ions when the concentration exceeds 2.0 m. The remaining Li ions readily coordinate with the B─F group, proving that ODFB^−^ acts as a MAB at high concentrations. Additionally, there is no significant alteration in the position and number of peaks as the temperature increases. The peaks at 890 and 910 cm^−1^ exhibit a marginal change of less than 4%, suggesting that the solvation structure of the electrolyte remains unaltered with variations in temperature (Figure S11, Supporting Information).

Based on the NMR and Raman spectra analysis, the solvation structure evolution in LiODFB electrolyte with increasing concentration is illustrated in Figure 1h. At low concentrations, the solvation sheath of Li ion primarily consists of SSIP. However, at a concentration of 2.0 m, the dominant structures shift to CIP and AGG. With increasing concentration, each C═O group of ODFB^−^ forms complexes with Li ions, leading to the transformation of ODFB^−^ into a MAB. The B─F functional group in the anion coordinates other free Li^+^ and contributes to the formation of a distinctive solvated structure, which increases the number of coordinated Li^+^ in one solvation sheath. Consequently, this unique solvation structure facilitates an increased Li^+^ transference number (Figures S12–S16, Supporting Information).

### 2.2 Performance of MCMB Anode at High Temperature

To further explore the impact of diverse solvation structures on the formation process of SEI film, we select the mesocarbon microbeads (MCMB) anode as a half‐cell model due to its widespread usage and high stability.

**Figure 2:**
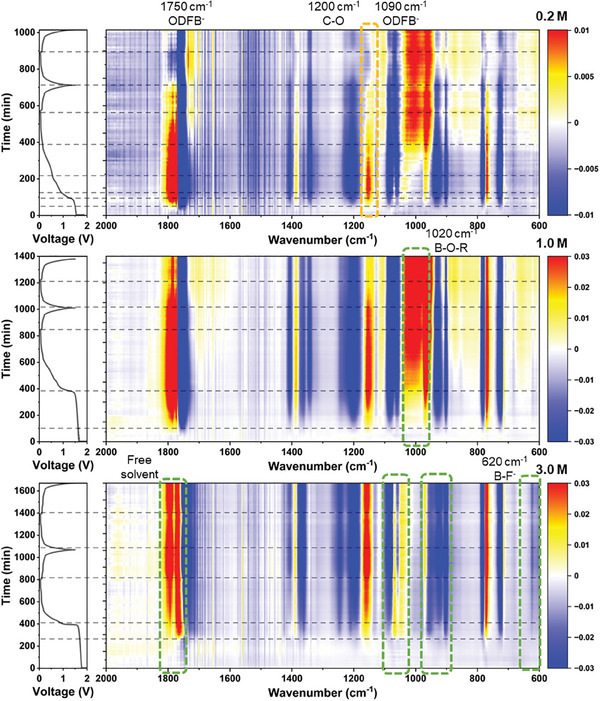
Dynamic evolution of electrolyte at the MCMB anode interface studied by in situ FTIR spectroscopy. Voltage–time curves and corresponding contour map of the FTIR spectra during the charge/discharge process when using the 0.2, 1.0, and 3.0 m electrolytes.

Characterizing the dynamic (de‐)solvation behaviors of each type of electrolyte at the anode interface is further conducted by in situ Fourier Transform Infrared (FTIR) spectroscopy, in addition to examining the solvation structure of different concentrations of the electrolytes (**Figure** **2** and Figures S17–S19, Supporting Information). The color bar, from blue to dark red, represents the gradually increased signal intensity of the electrolyte species at the anode side. The in situ spectral changes for the 0.2 and 1.0 m electrolytes exhibit similarity, albeit with a stronger overall signal for the 1.0 m electrolyte. In the prior part of the first discharge plateau, the spectral signal remains largely unchanged, and it increases with LiODFB concentration. The peak at approximately 1750 cm^−1^ corresponds to the C═O antisymmetric signal of the ODFB^−^, which exhibits the most negative signal and widest peak width during its transition from the 1.6 V platform to its ending. Promptly following the reduction of ODFB^−^, the free solvent peak at approximately 1780 cm^−1^ displays the most robust positive signal, concurrently attaining the broadest peak width at the end of the 1.6 V platform.^[^ ^20^ ^]^ This occurrence is attributed to the initial reduction of the ODFB^−^ coordinated to Li^+^, after which the proportion of free solvent in the electrolyte experienced an increase. The peak at its widest is also corroborated by the trends of the signals from the two sets of peaks at 770 cm^−1^ (free solvent) and 780 cm^−1^ (solvated peak), as well as 715 cm^−1^ (free solvent) and 725 cm^−1^ (solvated peak) (Figures S20 and S21, Supporting Information) The reversibility is not completely preserved during charging, as a substantial amount of solvent in the solvent is decomposed. Consequently, in the second cycle of the 0.2 m electrolyte, this region registers a negative signal, while a small residue is observed in the 1.0 m electrolyte. This finding suggests that an increase in LiODFB concentration can mitigate the consumption of solvent molecules. Moreover, the signal peak at 1780 cm^−1^ starts to weaken and narrow following the ending of the 1.6 V platform, suggesting the initiation of solvent consumption in the electrolyte. This reduction accelerates rapidly after the potential reaches 0.8 V, corresponding to the reduction of solvent molecules. The peak around 1160 cm^−1^ (*v*(C─O) of solvent) initially exhibits a similar trend. The regions at 969 cm^−1^ and 1010 cm^−1^ are attributed to the C─C peaks of EC/PC, while the interval of 980–1050 cm^−1^ corresponds to the B─O─R produced by the reduction of the ODFB^−^.^[^ ^20^, ^21^ ^]^ The signal strength of the 1.0 m solution is notably more robust than that of the 0.2 m solution. Overall, the changes in the electrolytes during charge and discharge, at both 0.2 and 1.0 m concentrations, are analogous, with no new peaks emerging or disappearing. This consistency indicates that ODFB^−^ behaves identically at low concentrations.

The in situ spectra of the high‐concentration electrolyte from 3.0 m exhibit interesting and prominent alterations. Firstly, despite the similarity in the duration of the two platforms, the spectral transformation time for 3.0 m is notably longer than that of 1.0 m, potentially suggesting an alteration in the LiODFB reduction reaction mechanism. Moreover, the signal intensity of the C═O at 1750 cm^−1^ is weaker than that of the 1.0 m electrolyte. If the decomposition approach is maintained at lower concentrations, the C═O in this region should exhibit a broader and more robust signal than that of 1.0 m, indicating a change in the way the C═O in ODFB^−^ participates in the reduction process within the high‐concentration LiODFB electrolyte. The peaks at 770, 780, 715, and 725 cm^−1^ exhibit a similar behavior to the other two concentrations, suggesting that ODFB^−^ molecules continue to be consumed. The peak of free solvent at 1780 cm^−1^ still displays a robust signal (positive), and it can change with the progression of charge and discharge, which distinguishes it from 0.2 and 1.0 m, indicating that the decomposition of free solvent in 3.0 m is inhibited. This finding reflects that a high concentration of electrolyte can promote the formation of a stable SEI film, thereby inhibiting the solvent reaction. The B─F peaks at 620 and 1090 cm^−1^ can be attributed to ODFB^−^, and the peaks at 1800 cm^−1^ can be ascribed to the formation of poly(carbonate), both of which occur concurrently. Liu's research demonstrates that in high‐concentration LiODFB electrolyte, the preference for breaking the B─F bond is evident, and B free radicals assault the solvent to generate poly(carbonate).^[^ ^22^ ^]^ This is consistent with the findings in the spectrum. Additionally, the region 980–1040 cm^−1^ exhibits a notable distinction from the electrolyte with a lower concentration, generating only fewer B─O─R products. This disparity reflects the variation in the reduction mode of ODFB^−^ at high or low concentrations and confirms the solvation structure in the electrolyte with high concentration, where ODFB^−^ becomes the MAB and binds to distinct solvation shells, which elicits a significantly distinct response.

**Figure 3:**
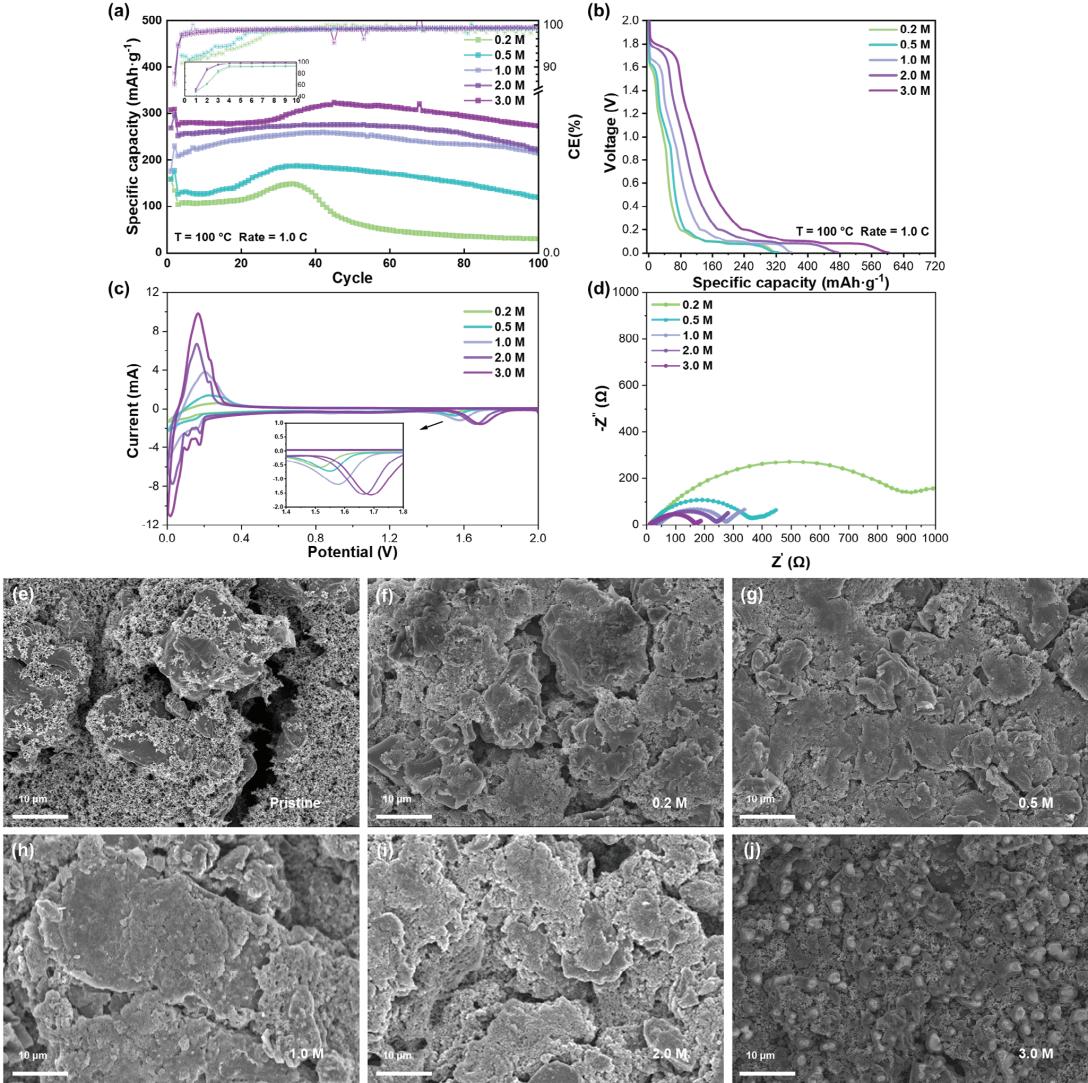
Electrochemical performance of Li/MCMB: a) Long cycling performance at a rate of 1.0 C at 100 °C. b) First discharge curves of Li/MCMB cells in different concentrations of the electrolytes. c) CV curves of Li/MCMB cells in different concentrations of the electrolytes under 0.1 mV s^−1^ at 100 °C. d) Impedance results of Li/MCMB cells after 100 cycles at 100 °C. e–j) SEM images of MCMB anodes after 100 cycles at 100 °C.

Additionally, coin cells with varying concentrations of the electrolytes are assembled to evaluate the battery performance under rigid high‐temperature conditions. At 100 °C, which is an extremely high temperature, all the batteries exhibit a distinct downward trend in **Figure** **3**a. The initial battery capacity gradually increases with the electrolyte concentration, which may be attributed to the enhanced conductivity of high‐concentration electrolytes at high temperatures and the Li^+^ transference number (Figures S12–S16 and S22, Supporting Information). Furthermore, the half‐cell exhibits a discernible increase in capacity during the initial cycles, which can be attributed to the widening of graphite layers upon cycling, resulting in decreased impedance. This phenomenon is expected to be more pronounced under elevated temperatures.^[^ ^23^ ^]^ In particular, for the electrolyte concentration of 0.2 m, high temperatures can significantly increase the consumption of the electrolyte, leading to the most dramatic reduction in the battery capacity. The first discharge curve of the battery in varying concentrations of the electrolytes is depicted in Figure 3b. Like the curve observed through in‐situ infrared analysis, an initial plateau emerges at approximately 1.8 V, which is attributed to the decomposition of ODFB^−^. This reaction promotes the formation of a robust SEI layer, effectively preventing direct contact between the solvent and electrode surface, which inhibits the over‐decomposition of the solvent. The disappearance of EC/PC decomposition platforms located at about 0.8 V at high concentrations further supports this finding.^[^ ^24^ ^]^ Moreover, the impedance of the battery also exhibits a correlation with its capacity. After 100 cycles, the higher impedance observed at low concentrations can be attributed to the occurrence of a pronounced side reaction resulting from direct contact between solvent molecules and the electrode surface at high temperatures. This leads to an increase in organic byproducts, consequently elevating the impedance. Conversely, the significantly lower impedance observed for 3.0 m concentration suggests that HCE facilitates the formation of a highly conductive and stable SEI layer, thereby enhancing cell performance under harsh high‐temperature conditions (Figure 3d). The CV curve also exhibits reduction peaks at approximately 1.6 V, along with symmetrical oxidation/reduction peaks below 0.5 V, which provide evidence for the formation of the solid electrolyte interphase (SEI) layer during the first cycle. Moreover, an increase in LiODFB concentration leads to a more pronounced decomposition peak of ODFB^−^ that shifts towards higher potentials, consistent with observations from the initial discharge cycle curve (Figure 3c).

Furthermore, scanning electron microscopy (SEM) is used to examine the morphology of Li/MCMB cells cycled at a temperature of 100 °C. As depicted in Figure 3e–j, no significant disparities are observed in the micrographs for electrolyte concentrations below 2.0 m. However, when the electrode is subjected to cycling with a concentration of 3.0 m LiODFB, numerous inorganic small spherical particles are detected on its surface, indicating a notable contrast between this concentration and others. This discrepancy suggests that different reactions occur under higher electrolyte concentrations leading to the formation of these products and their subsequent deposition onto the electrodes.

**Figure 4:**
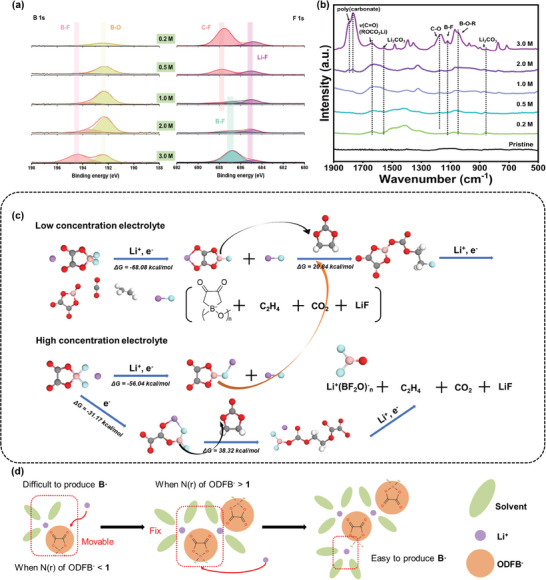
a) XPS profiles for the MCMB anode cycled in various electrolyte concentrations including B 1s and F 1s. b) FTIR spectra of cycled MCMB anodes in investigated electrolyte concentrations. c) Reduction process of low‐concentration electrolyte and high‐concentration electrolyte. d) Schematic illustrating the solvation structure evolution and reaction model.

The chemical composition of the SEI layer is measured by X‐ray photoelectron spectroscopy (XPS) as shown in **Figure** **4**a and Figure S23 (Supporting Information). In case of a low‐concentration electrolyte (0.2 and 0.5 m) the C─F peak is located at 688 eV on the cycled electrode surface, as evidenced by the presence of C 1s peak (C─F, 290.0 eV) in the spectrum, which is possibly attributed to the reaction between PVDF binder and the solvent, resulting in its precipitation at high temperatures and subsequently affecting the stability of the electrode surface and rise the interface impedance.^[^ ^9^, ^25^ ^]^ When the concentration of LiODFB increases, the peak of C─F gradually disappears, while the peak of B─F gradually appears, which can be attributed to the initial attack of a Li ion on one of the B─F bonds in the ODFB^−^ to produce LiF.^[^ ^22^ ^]^ Subsequently, the ODFB^−^ anion ring opens and forms BOF free radicals that further attack other solvents, leading to polymer carbonate formation.^[^ ^21^, ^26^ ^]^ Moreover, the B 1s spectrum demonstrates that the product remains essentially unaltered at concentrations below 1.0 m; however, at concentrations exceeding 2.0 m, a significant abundance of products containing B─F are formed on the electrode surface. This observation further substantiates the presence of a distinctive solvation structure in highly concentrated electrolytes, wherein ODFB^−^ acts as an MAB connected to multiple distinct solvation sheaths. To further validate this result, we use the DFT method to compute the desolvation energy and LUMO–HOMO of representative solvation structures in concentrated LiODFB electrolytes (Figure S24, Supporting Information). When Li^+^ is coordinated with the F atom, the overall LUMO exhibits a decreased magnitude, rendering the reduction reaction more favorable. Additionally, the desolvation energy decreases, promoting the reaction and yielding a substantial number of free radicals. The conclusions are further supported by the ATR (attenuated total refraction)‐FTIR spectra of the electrode surface after cycling (Figure 4b). At a concentration of 0.2 m, the inability to form a stable SEI film on the electrode surface leads to direct contact between solvent molecules and the electrode, resulting in violent decomposition and generation of numerous solvent‐decomposed products. With increasing concentration, there is no significant change in surface products due to minimal alteration in solvation structure. However, when the concentration reaches 3.0 m, there is a sharp increase in the strength of surface functional groups accompanied by substantial formation of poly(carbonate) (1800 cm^−1^), Li_2_CO_3_ (1500 cm^−1^), as well as involvement of B radical in reactions (B─F at 1090 cm^−1^; B─O─R at 1020 cm^−1^).^[^ ^21^, ^26^, ^27^ ^]^

Furthermore, DFT is also used to provide additional evidence for the reduction reaction pathway (Figure 4c and Figures S24 and S25, Supporting Information). At low concentrations, Li ions are found to preferentially form complexes with two carbonyl groups on ODFB^−^, as supported by previous MD, Raman and NMR results. Upon obtaining a Li^+^ and an e^−^, an F atom dissociates from the B atom, resulting in the formation of LiF and an active structure containing a B free radical. This structure exhibits a tendency to attack solvent molecules such as EC, leading to ring‐opening reactions of the solvent molecules.^[^ ^21^, ^28^ ^]^ Subsequently, further decomposition of lithium ions and electrons generates anionic polymers along with ethylene, carbon dioxide, and LiF.^[^ ^29^ ^]^ At high concentrations, during the first stage transition state, there are two forms observed; however, only the second form gives rise to a distinct B free radical structure different from that at low concentrations. The resulting product is identified as (BF_2_O) *_n_* ^−^ free radicals which can continue attacking solvents to produce polymer carbonate species consistent with FTIR and XPS observations mentioned above. Notably, regardless of electrolyte concentration being low or high, the amount of generated LiF remains similar; hence explaining why there is no significant peak change observed for Li─F in XPS analysis, while the intensity of B─F peak is growing above 2.0 m electrolyte.

Therefore, LiODFB salts may exhibit distinct decomposition mechanisms at varying concentrations, thereby influencing the properties of the SEI layer (Figure 4d). At low concentrations, the average solvated structure of the electrolyte contains less than 1 ODFB^−^, indicating a limited presence of CIP structures and minimal formation of AGG structures in the solution. However, within this structure, due to the higher electronegativity of C═O compared to B─F, lithium ions still preferentially complex with stronger C═O sites during solvation sheath reduction, resulting in similar products. Once the concentration reaches a certain threshold (2.0 m in this study), the proportion of ODFB^−^ exceeds 1 in the solvated structure leading to definite AGG formation. However, once AGG is formed and strong binding sites are occupied by Li ions, and Li ions tend to complex with B─F sites instead. Consequently, ODFB^−^ anions transform into MAB species that bridge two different solvation sheaths and facilitate more B radical generation for attacking and producing abundant polymer carbonates. This process leads to the formation of a more stable SEI film that enhances battery cycling stability.

### 2.3 Performance of Cathode at High Temperature

To explore the difference of oxidation process from different solvation structure in varying concentrations, Li/LiFePO_4_ (LFP) half‐cells are assembled to observe the cathode electrolyte interphase (CEI) layer formation at 100 °C.

**Figure 5:**
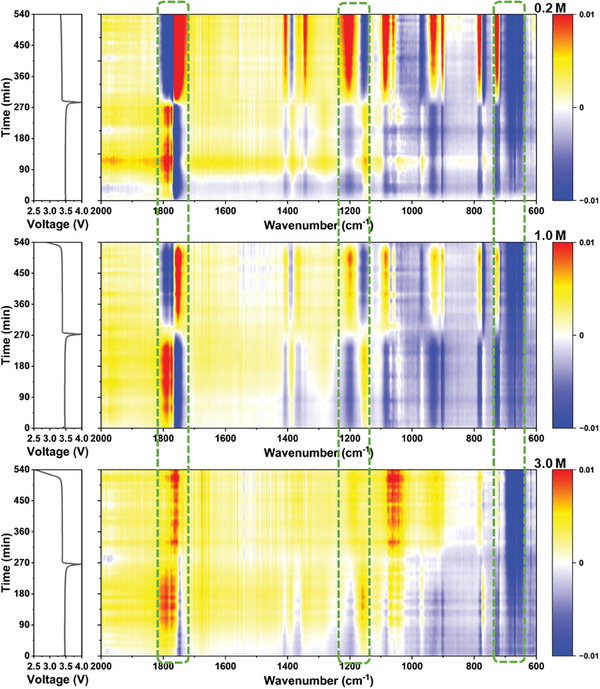
Dynamic evolution of electrolyte at the LFP cathode interface studied by in situ FTIR spectroscopy. Voltage–time curves and corresponding contour map of the FTIR spectra during the charge/discharge process when using the 0.2, 1.0, and 3.0 m electrolytes.

The in situ FITR spectra are also utilized to further elucidate the dynamic solvation and desolvation phenomena occurring at the cathode interface for each concentration of electrolyte (**Figure** **5**). Due to the low conductivity of the dilute electrolyte, a substantial polarization voltage is observed during the initial charge and discharge of the half‐cell; however, subsequent charging and discharging proceeded normally (Figures S26–S28, Supporting Information). For a low‐concentration electrolyte of 0.2 m, there is a clear negative signal at the position attributed to the free EC/PC solvent during charging around 1780 cm^−1^, which indicates that the solvent molecules are consumed in large quantities during the first cycle. However, after a period of discharge, a strong positive signal appears at the position of 1800 cm^−1^, which is attributed to the Li ion in the positive electrode and the free solvent molecules in the solution to form the structure of Li^+^–solvent. After the first discharge, the Li^+^–solvent decreases sharply, and the free solvent molecules increase significantly.^[^ ^30^ ^]^ Additionally, the increase in LiODFB concentration results in a reduced abundance of free solvents within the 1.0 m electrolyte, leading to a comparatively weaker overall signal change compared to lower concentrations. However, the number of peaks remains similar. In the case of the 3.0 m electrolyte, intriguing phenomena manifest themselves. Due to the scarcity of free solvent molecules at high concentrations, the depletion of the free molecular signal during the initial charging cycle is significantly less pronounced compared to that observed at 0.2 and 1.0 m concentrations. Furthermore, unlike in low‐concentration electrolytes, there is no discernible enhancement in signal intensity for 1800 cm^−1^ in high‐concentration electrolytes during early stages of charging, suggesting that Li^+^ extracted from the cathode do not coordinate with the solvent molecules. Following the charging duration of 180 min, a broader red zone emerges relative to Li^+^–solvent, potentially attributed to the formation of poly(carbonate). Interestingly, even after discharge, although slightly shifted, the signal at around 1800 cm^−1^ remains red, indicating an increased presence of Li^+^–solvent. This finding can be potentially attributed to the consumption of a certain amount of ODFB^−^ during the initial charging cycle resulting in irreversible formation of CEI layer while leaving Li^+^ unconsumed and enabling re‐complexation with solvent molecules. Thus, the solvation structure in high concentration of LiODFB electrolyte also exhibits unique dynamic effects.

**Figure 6:**
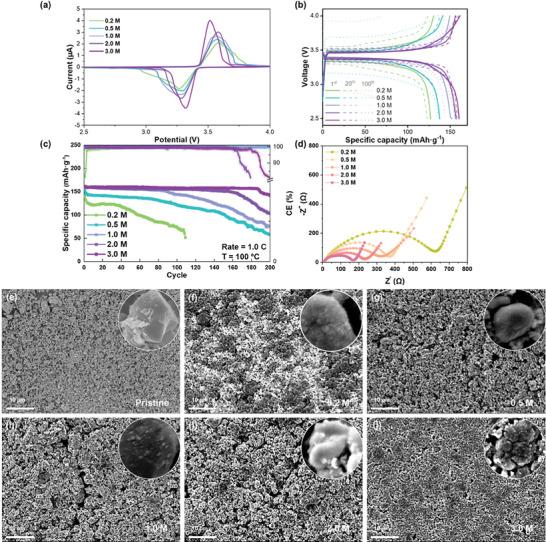
Electrochemical performance of Li/LFP a) CV curves of Li/LFP cells in different electrolyte concentrations under 0.1 mV s^−1^ at 100 °C. b) First discharge curves of Li/LFP cells in different electrolyte concentrations. c) Long cycling performance at a rate of 1.0 C at 100 °C. d) Impedance results of Li/LFP cells after 100 cycles at 100 °C. e–j) SEM images of LFP cathodes after 100 cycles at 100 °C.

The CV curves of Li/LFP half‐cells at a scan rate of 0.1 mV s^−1^ and 100 °C are presented in **Figure** **6**a. At low concentrations, the oxidation and reduction peaks of LFP exhibit a wide and short shape, indicating limited kinetic properties due to the low conductivity of the electrolyte at low concentrations. As the concentration increases, there is a gradual increase in peak current accompanied by a decrease in half‐peak width, suggesting lowered polarization and improved charge‐transfer ability of the electrolyte.^[^ ^31^ ^]^ The electrochemical performance of LFP half‐cells with different concentrations of the electrolytes at rates of 1.0 and 5.0 C is depicted in Figure 6b,c and Figure S29 (Supporting Information), respectively. The initial capacity of the LFP half‐cells also follows the trend observed in the MCMB half‐cells, suggesting that it is influenced by both the electrolyte conductivity and the Li^+^ transference number. Additionally, the battery's impedance demonstrates a positive correlation with its capacity, indicating that a high‐concentration electrolyte facilitates the formation of a stable and highly conductive CEI film (Figure 6d). Notably, the cycle stability of the 1.0 C cycle battery is enhanced with increasing concentration. However, it should be noted that batteries with concentrations exceeding 2.0 m exhibit a distinct decay pattern compared to those with lower concentrations. The former demonstrates a gradual decline after a certain number of cycles, accompanied by a sharp decrease in efficiency, which may be attributed to the formation of numerous polymer carbonates at higher concentrations. Conversely, no efficiency degradation is observed at lower electrolyte concentrations; instead, the capacity reduction is ascribed to continuous electrolyte consumption. However, this phenomenon is not replicated at high‐rate cycles due to the predominant presence of Li_2_CO_3_’s internal inorganic layer as decomposition products under elevated temperatures and range, which differs from that observed at lower currents (Figures S30–S34, Supporting Information).^[^ ^32^ ^]^

The morphology of cycled LFP electrodes in different concentrations of the electrolytes is obtained by SEM (Figure 6e–j). The LFP with 3.0 m electrolyte exhibits a most smooth and dense morphology, and the secondary particles are completely covered by a dense CEI layer. In contrast, the electrolytic surface with a 0.2 m concentration electrolyte is coated with a layer of substances exhibiting low conductivity, which can be attributed to the intense decomposition of free solvent molecules in the diluted electrolyte at elevated temperatures, resulting in an abundance of decomposition products derived from organic solvents. The morphologies of other concentrations exhibit a tendency towards similarity, thereby indicating that the formation of stable CEI films is influenced by the distinctive solvation structure established by the electrolyte at specific concentration thresholds.

**Figure 7:**
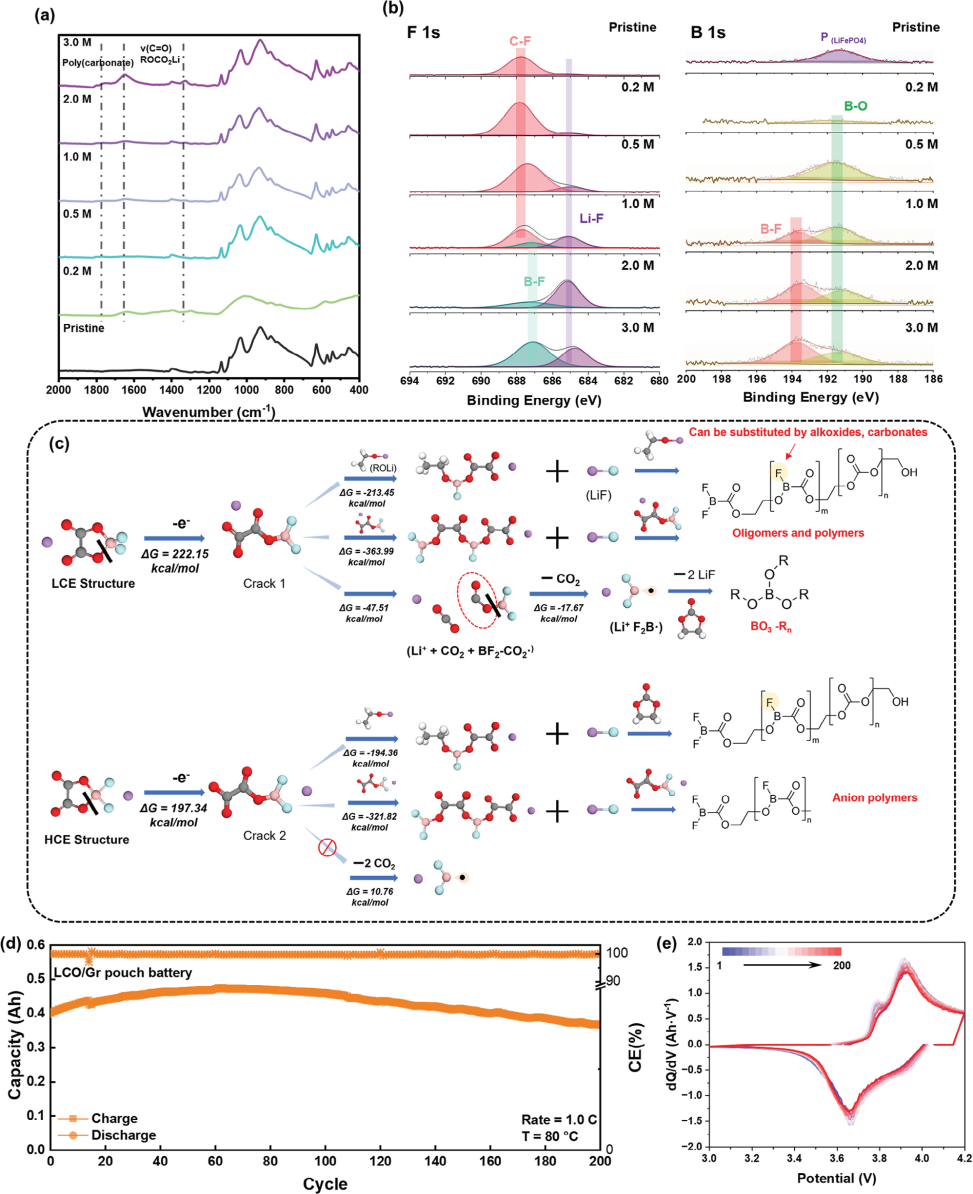
a) FTIR spectra of cycled LFP cathodes in investigated electrolyte concentrations. b) XPS profiles for the LFP cathode cycled in various electrolyte concentrations including B 1s and F 1s. c) Oxidation process of low‐concentration electrolyte and high‐concentration electrolyte. d) Long‐term performance of LCO/Gr pouch cell in high temperature after activation process. e) Differential capacity (d*Q*/d*V*) plots of the 1–200th charge/discharge curves of LCO/Gr pouch cell.

In order to investigate the chemical composition of the CEI film on the surface of the positive electrode, the LFP positive electrode is analyzed using ATR and XPS after different electrolyte cycles (**Figure** **7**a,b). The distinction between products generated at the surface of LFP is less discernible compared to those formed on the anode side. The intensity at 1750, 1650, and 1300 cm^−1^ gradually increases, which is attributed to poly(carbonate), R‐OCO_2_Li, and Li_2_CO_3_, respectively, indicating that the decomposition of LiODFB can promote the formation of more inorganic CEI films. The distribution of CEI components in different concentrations of the electrolytes is illustrated in Figure 7b. In contrast to the negative side, LiF is not observed at low concentrations on the positive side, while the B─F trend follows a similar pattern as the negative side, gradually decreasing until it disappears at 2.0 m. The B1s spectrum reveals a minimal presence of B─O at 0.2 m. In the C 1s spectrum, except for 0.2 m, the distribution of peak patterns remains highly similar; however, an increase in concentration leads to a stronger presence of O─C═O (Figure S35, Supporting Information). These findings collectively suggest that the electrolyte exhibits distinct reactions at the positive and negative electrodes, with a particular emphasis on the oxidation of ODFB^−^ to form a rich‐inorganic CEI film, which effectively suppresses excessive solvent decomposition.

Figure 7c illustrates the potential reaction pathways at varying concentrations. Upon electron loss during the initial step of ring‐opening reaction, the HCE structure exhibits a lower energy barrier and is more prone to undergo ring‐opening. Consequently, the Li^+^ coordinated with the F atom demonstrates a higher HOMO, which is more susceptible to oxidation (Figure S24, Supporting Information) It has a greater tendency to lose electrons on the cathode surface. The resulting ring‐opened LiODFB generates electron‐deficient free radicals, which subsequently react with electron‐rich alkoxides, leading to the formation of (BOR─O─R─O) *_n_*—polymer components resembling those observed previously. Alternatively, another possibility arises where these free radicals persist in attacking anions and generate borate polymer free radicals that can further interact with solvents or alkoxides to produce, wherein F may be substituted by alkoxides and carbonates when the concentration of anion is low. The third pathway involves the decomposition of an anion, producing Li^+^BF_2_· through the elimination of two carbon dioxide molecules.^[^ ^33^ ^]^ Subsequently, this radical can react with solvent molecules to generate tri‐coordinated B due to high concentration of EC/PC solvent.^[^ ^21^ ^]^ However, at high concentrations, the spontaneous formation of Li^+^BF_2_· is hindered by positive Gibbs free energy, leading to a reduction in B─O content at the interface. Additionally, a significant number of anions undergo ring‐opening to generate free radicals, resulting in an augmented total polymer(carbonate) and oligomers. This also leads to a substantial disparity between the quantities of “m” and “n” in the products of the first pathway (*m* > *n*), consequently elevating the presence of inorganic compounds within CEI and diminishing interface impedance. In summary, during the discharge process, the solvation structure of the high‐concentration electrolyte undergoes electron and Li^+^ transfer, leading to the generation of B free radicals that react with solvent molecules, resulting in a substantial formation of polymer carbonates. During the charging process, the initial reaction involves ring‐opening of ODFB^−^ followed by subsequent reactions yielding oligomers, polymers, and borates.

Furthermore, a commercial full pouch cell (LiCoO_2_||Gr, 0.5 Ah, 4.2 V, N/P = 1.15/1) is used to verify the excellent performance of 3.0 m electrolyte at high temperature. Figure S36 (Supporting Information) demonstrates the similarity in SEI composition between MCMB and graphite anodes. All the peaks are almost matching, thereby indicating the reproducibility of this approach. Figure 7d and Figure S37 (Supporting Information) show the incredibly good high‐temperature cycle stability of industrial full cells. The activated battery has a capacity of 0.4 Ah on the first cycle and peaked at 0.48 Ah on the 60^th^ cycle, and demonstrated a capacity retention of 82.4% after 200 cycles. The gradual decline followed by an exceptionally high average efficiency of 99.74% suggests that the high‐concentration electrolyte retains a commendable adaptability for commercial pouch batteries (Table S1, Supporting Information).^[^ ^30^, ^34^ ^]^ Figure 7e displays that the electrode material still shows good reversibility when cycling at high temperature. The SEI and CEI films generated by the reaction of its unique solvation structure facilitate a stable battery circulation under high temperatures. Additionally, the cycling performance of NCM523||Gr (0.5 Ah, 4.1 V, N/P = 1.15/1) at higher temperature at 100 °C is tested (Figure S38, Supporting Information), which could achieve a retention of 80.0% after 50 cycles suggests that this electrolyte with special solvation structure can support the operation of pouch batteries in extremely high‐temperature environments.

## 3 Conclusion

In summary, we report an innovative concept named multipoint anionic bridge that chooses a kind of lithium salt in which the anion has several kinds of coordinate points to connect within different solvation structures, like LiODFB. In situ FTIR is used to observe the evolution of the charge–discharge process in Li/MCMB and Li/LFP with different concentration of the electrolytes. In the high‐concentration electrolyte, the ASS at the anode side facilitates preferential generation of B free radicals by anions, attacking the solvents to promote polycarbonate reaction to form the stable SEI layer. At the cathode side, the MAB‐connected ASS has a higher HOMO, promoting a preferential oxidation process to form the borates polymeric organic phase by anions, thereby stabilizing the cycle on the positive side. However, this phenomenon is less pronounced compared to that observed on the negative side. Moreover, high‐concentration electrolytes achieve a very high‐capacity retention (82.4%, 200 cycles) at 80 °C (CE > 99.7%) and retention (80.0%, 50 cycles) at 100 °C for commercial pouch cells. This work paves the way for the development of high‐performance electrolyte systems by designing and using new multipoint anions to construct asymmetric solvation structures.

## Conflict of Interest

The authors declare no conflict of interest.

## Supporting information



Supporting Information

## Data Availability

The data that support the findings of this study are available from the corresponding author upon reasonable request.
