# Supplementary material for: Multipoint Anionic Bridge: Asymmetric Solvation Structure Improves the Stability of Lithium‐Ion Batteries
Source: Adv Sci (Weinh). 2024 Oct 30;11(48):2410329. doi: 10.1002/advs.202410329 (PMC11672286; doi:10.1002/advs.202410329)
Supplement: Supplementary file 1 — Supporting Information [file ADVS-11-2410329-s001.pdf]

ADVANCED SCIENCE
Open Access

# Supporting Information

for *Adv. Sci.*, DOI 10.1002/advs.202410329

Multipoint Anionic Bridge: Asymmetric Solvation Structure Improves the Stability of Lithium-Ion Batteries

*Tianle Zheng, Tonghui Xu, Jianwei Xiong, Weiping Xie, Mengqi Wu, Ying Yu, Zhuijun Xu, Yuxin Liang, Can Liao, Xiaoli Dong, Yongyao Xia, Ya-Jun Cheng*, Yonggao Xia* and Peter Müller-Buschbaum**

# Multipoint Anionic Bridge: Asymmetric Solvation Structure Improves the Stability of Lithium-Ion Batteries

*Tianle Zheng, Tonghui Xu, Jianwei Xiong, Weiping Xie, Mengqi Wu, Ying Yu, Zhuijun Xu, Yuxin Liang, Can Liao, Xiaoli Dong, Yongyao Xia, Ya-Jun Cheng,* Yonggao Xia,* Peter Müller-Buschbaum**

## Experimental Procedures

### Materials

The materials were used as received without any additional purification treatment. Poly(vinylidene fluoride) (PVDF) was obtained from Solvay. N-methyl-2-pyrrolidone (NMP, purity of 99.0%), ethylene carbonate (EC, purity of 99%), dimethyl carbonate (DMC, purity of 99%) and propylene carbonate (PC, purity of 99%), were purchased from Aladdin Reagent Co., Ltd., China. Conductive carbon black (Super P) was obtained from SCM Chem in Shanghai, China. Lithium hexafluoride phosphate ($LiPF_6$) and lithium difluoro(oxalate)borate (LiODFB), both with a purity exceeding 99.9% and containing less than 10 ppm of $H_2O$, were procured from Zhangjiagang Guotai-Huarong Commercial New Material Co., Ltd. The lithium foil, approximately 300 μm thick, was sourced from Dongguan Shanshan Battery Materials Co., Ltd. A commercial electrolyte (CE), consisting of a 1.0 M $LiPF_6$ solution in EC/DMC (3:7 wt./wt.), was sourced from Guotai-Huarong Commercial New Material Co., Ltd. The $LiFePO_4$ (LFP) powder was procured from LeNeng Co., Ltd in China, while the mesocarbon microbeads (MCMB) used in this work were donated by Veken. The industrial pouch cells were donated from Shenzhen Utility Energy CO., LTD.

### Electrolyte solution Preparation and Electrode Fabrication

The present study involved the preparation of five distinct concentrations of electrolytes: 0.2 M, 0.5 M, 1.0 M, 2.0 M, and 3.0 M LiODFB dissolved in an EC/PC (1:1 v/v) mixture solution under continuous stirring at a temperature of 60 °C for a duration of two hours with a rotational speed set at 1000 rpm within a controlled environment glove box. The LFP and MCMB powders were dried at 80 °C under vacuum for 8 hours prior to electrode fabrication. Subsequently, the dried powders were mixed with super P and PVDF powders in a mass ratio of 8:1:1 in NMP. Continuous stirring was conducted for 12 hours to ensure complete dissolution of PVDF and homogeneous powder mixing. The resulting slurries were then cast onto Al or Cu current collectors with mass loading densities ranging from approximately 5.0 to 7.0 mg·cm$^{-2}$ relative to the active material.

**Electrochemical Tests**

Li||LFP and Li||MCMB cells were all assembled in CR 2032-type coin cells with 80 μL of the specific electrolyte solution. The industrial NMC523||graphite pouch batteries with a total capacity of 0.5 Ah were injected with 1.5 ml electrolyte (N/P ratio = 1.15/1). The industrial LCO||graphite pouch batteries with a total capacity of 0.5 Ah were injected with 1.8 ml electrolyte (N/P ratio = 1.15/1). Each cell assembling and electrolytes injection were operated in a glove box (MBRAUN UniLab) with $O_2$ and $H_2O$ level of less than 0.1 ppm.

Long-term cycling and rate tests were performed on the Li||LFP battery with a voltage range of 2.50 V to 4.00 V (vs. $Li^+$/Li) and the Li||MCMB battery with a voltage range of 0.005 V to 2.0 V (vs. $Li^+$/Li). To investigate the electrolyte evolution and reactions at extremely high temperatures (100 °C) on both sides of the cathode and anode, Li||LFP half cells with varying concentrations of electrolyte were cycled at 1.0 C (1.0 C = 170 mAh·$g^{-1}$), while Li||MCMB half cells were cycled at 1.0 C (1.0 C = 372 mAh·$g^{-1}$) in an oven. LCO||Gr pouch cells were tested with a voltage range from 3.0 to 4.2 V (vs. $Li^+$/Li), and NCM523||Gr pouch cells were tested with a voltage range from 3.0 to 4.1 V (vs. $Li^+$/Li).

Cyclic voltammetry measurements were conducted using an electrochemical workstation (Solartron Analytical) in the voltage range of 2.50 V to 4.00 V for Li||LFP and from 0.005 V to 2.00 V for Li||MCMB, while the electrochemical impedance spectroscopy tests were performed at frequencies ranging from 0.01 Hz to 1 MHz with a voltage amplitude of 10 mV. Both Li||LFP and Li||MCMB batteries were subjected to testing at the discharge end.

In accordance with a previously reported methodology, the Li/Li symmetric cell architecture was employed to estimate the migration number of lithium ions ($t_{Li+}$) in the electrolyte. [1] EIS experiments were conducted both before and after the current reached a steady state by applying a minute polarization potential of 10 mV to the symmetric cell (with a relaxation time of 3000s). The following equation was utilized for calculating $t_{Li+}$.

$$t_{Li^+} = \frac{I_s(\Delta V - I_o R_o)}{I_o(\Delta V - I_s R_s)}$$

where $I_o$ and $I_s$ represent the current of the initial and stable state after polarization, respectively. $R_o$ and $R_s$ express the interfacial resistance values of the lithium electrode before and after polarization. $\Delta V$ is the polarization potential.

**Characterization of Electrodes and Electrolyte Solution**

Fourier transform infrared spectroscopy (FTIR, NICOLET 6700) analysis was performed at a temperature of 25 °C. Raman spectra of the electrolytes were obtained using a Renishaw in Via Reflex Raman microscopy system at

ambient temperature. Scanning electron microscopy (SEM) images were acquired from a Hitachi S4800 cold-field emission scanning electron microscope equipped with energy-dispersive X-ray spectroscopy (EDS). The X-ray photoelectron spectroscopy (XPS) measurements were conducted utilizing the Axis Ultra DLD XPS spectrometer. 1. The $^{7}$Li, $^{11}$B, and $^{19}$F nuclear magnetic resonance (NMR) experiments were conducted using an AVANCE NEO 600 NMR spectrometer. The electrolyte mass employed in the NMR experiments was approximately 600 ul. Before any characterization, all samples were safeguarded in containers filled with argon gas to prevent direct exposure to air.

**Evolution of in-situ ATR-FTIR experiment**

In-situ ATR-FTIR experiments were acquired with a Thermofisher-Nicolet 6700 FTIR spectrometer equipped with an electrochemical cell having two electrodes. The electrochemical cell used diamond crystal as the IR window. The cell is made of polyether ether ketone material (PEEK). All spectra were acquired with a resolution of 4 cm$^{-1}$. The spectra are presented in the form of absorbance $\boldsymbol{A = log(1/T) = -log(R_s/R_{ref})}$, where $R_s$ and $R_{ref}$ are the single-beam spectra of the sample charged state and open-voltage state, respectively.

**Calculation Method**

The molecular dynamics simulations were performed using the GRMOACS 2020.6 package. [2] The Visualization of structures was performed by VMD software. [3] The molecules were mixed in a cubic box with periodic boundary conditions by using PACKMOL package. [4]The Generation Amber Force Field (GAFF) was selected in this work, which is good for investigation of various small organic molecules. [5] The SOBTOP code was used to generate the desired force field parameters for the simulation systems. The partial charges on atoms were obtained using the restrained electrostatic potential (RESP) method, which was calculated with Multiwfn software. [6]

Before starting the MD simulation, the initial configurations were relaxed using a conjugate gradient minimization scheme. The step size was 0.01 nm, and the cycle was set to 5000 steps. The minimization was considered to have converged when the minimum force was less than 100 kJ·mol$^{-1}$·nm$^{-1}$. The van der Waals interaction was calculated by the cut-off method, atomic electrostatic interaction was calculated by PME (particle mesh Ewald), and both the cut-off and PME distances were 1.0 nm. [7] Then, the system was equilibrated with a pressure of 1.0 bar to achieve the desired density. The Berendsen and V-rescale methods were used to control the pressure and temperature. The time constant was 1.0 ps, and the compressibility was $4.5 \times 10^{-5}$ bar$^{-1}$. The equilibrium was 5 ns for all systems with a 0.001 ps time step. Finally, the production ran for 50 ns. The pressure control was changed to the Parrinello-Rahman method in the production run. In addition, the LINCS (Linear Constrain Solver) algorithm was used to impose constraints on the hydrogen bond. [8]

**Supporting Figures**

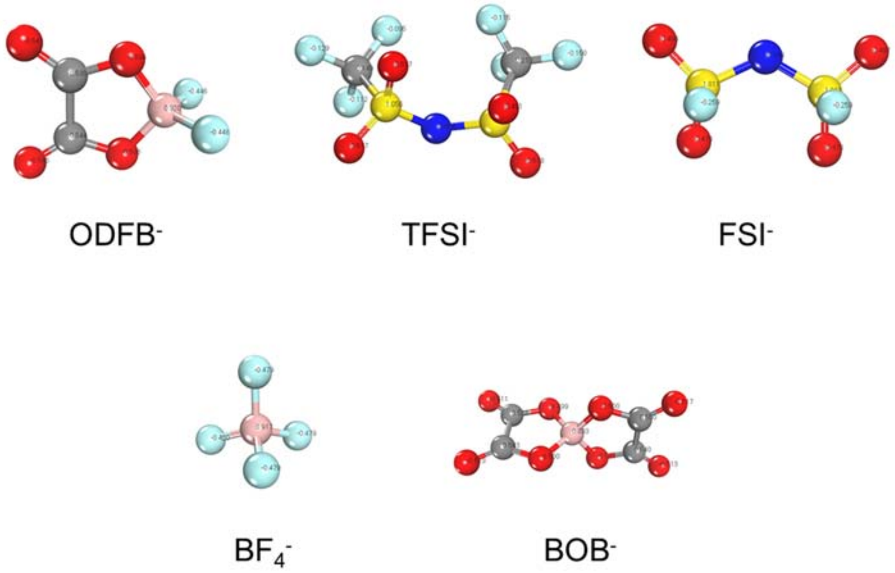


Figure S1. Molecular structures and charge distribution of different anions.

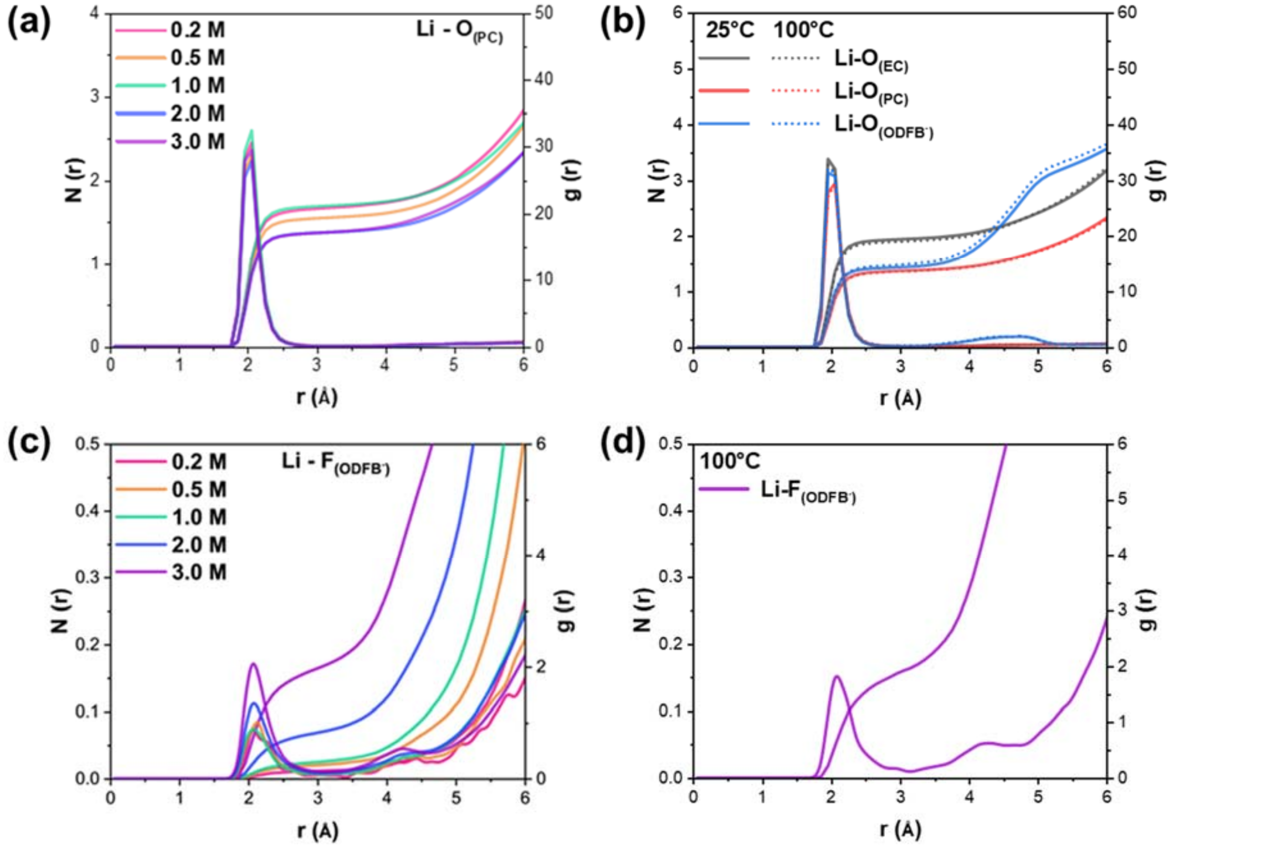


Figure S2. (a, c) RDF of Li – $O_{(PC)}$ and Li – $F_{(ODFB^-)}$ in various concentration electrolytes. (b, d) RDF of Li – $O_{(PC)}$ and Li – $F_{(ODFB^-)}$ in 3.0 M electrolyte at 25 and 100 °C.

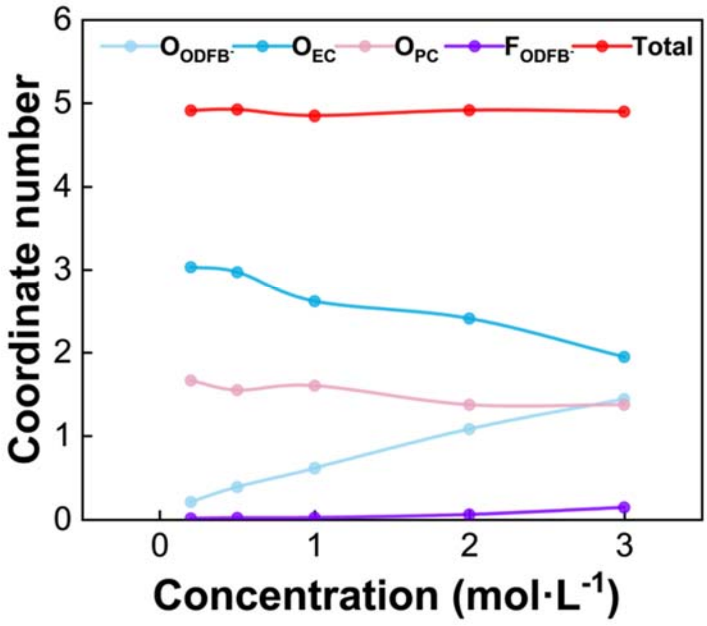


Figure S3. Evolution of coordination number in elevated concentration electrolytes.

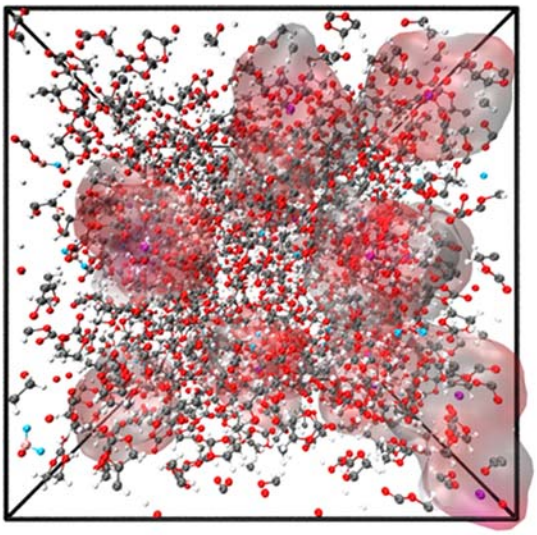

Figure S4. Snapshots of the solvated structure of 0.5 M concentration electrolyte.

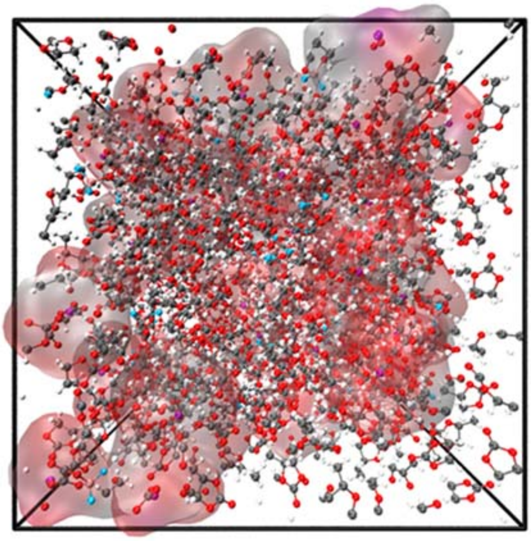

Figure S5. Snapshots of the solvated structure of 1.0 M concentration electrolyte.

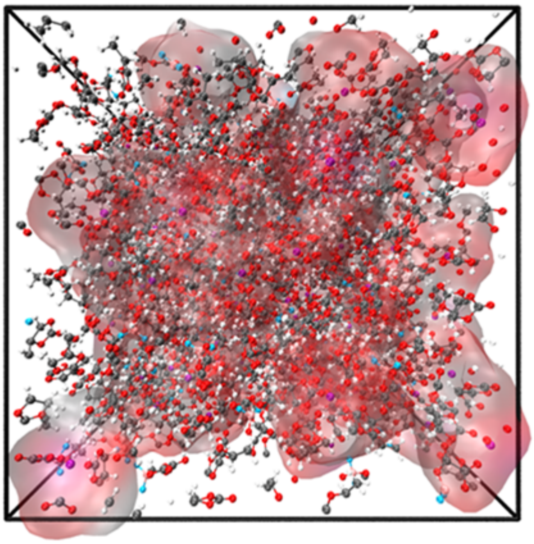

Figure S6. Snapshots of the solvated structure of 2.0 M concentration electrolyte.

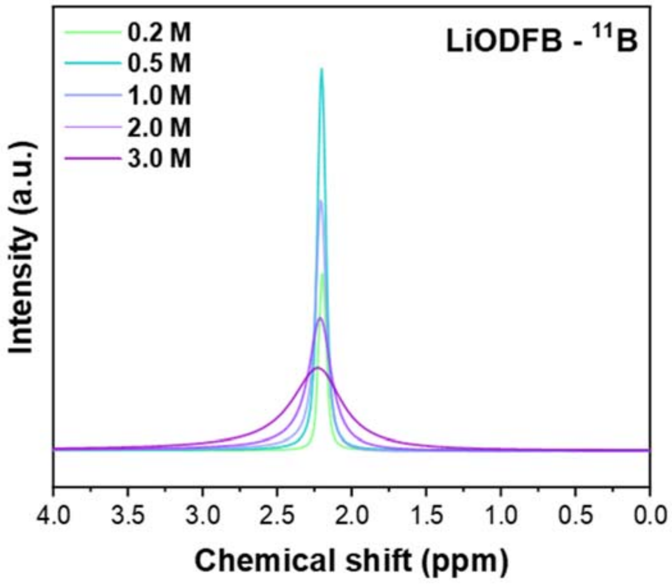


Figure S7. $^{11}B$ NMR spectra of different LiODFB electrolyte concentrations.

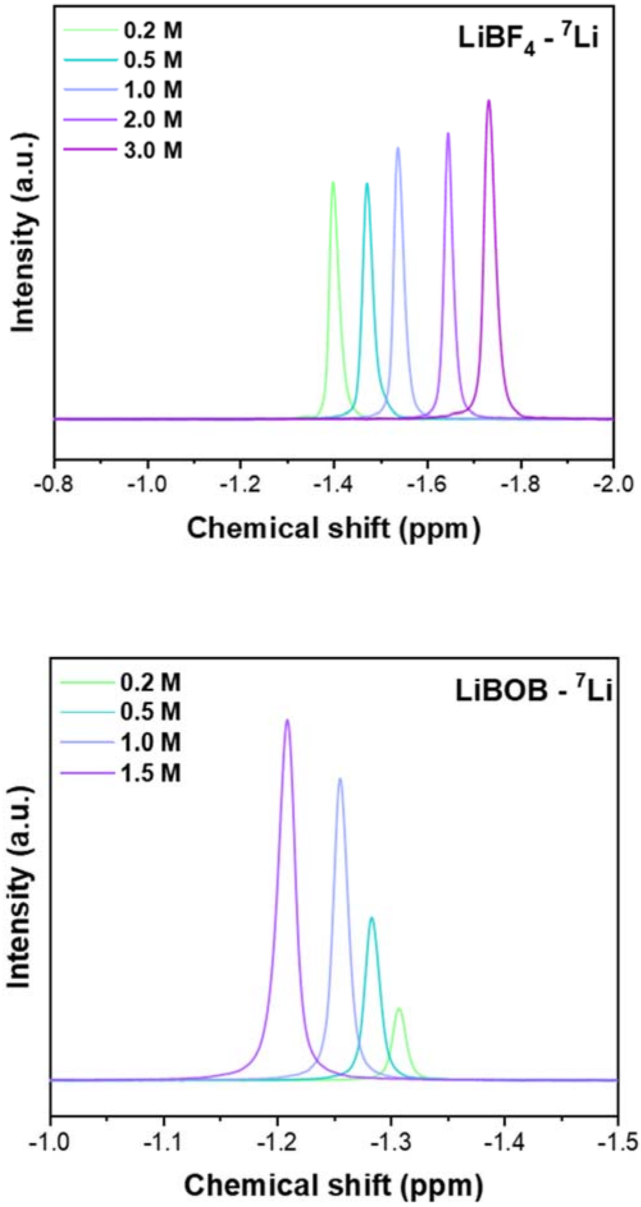


Figure S8. $^{7}Li$ NMR spectra of different electrolyte concentrations of $LiBF_4$ and LiBOB.

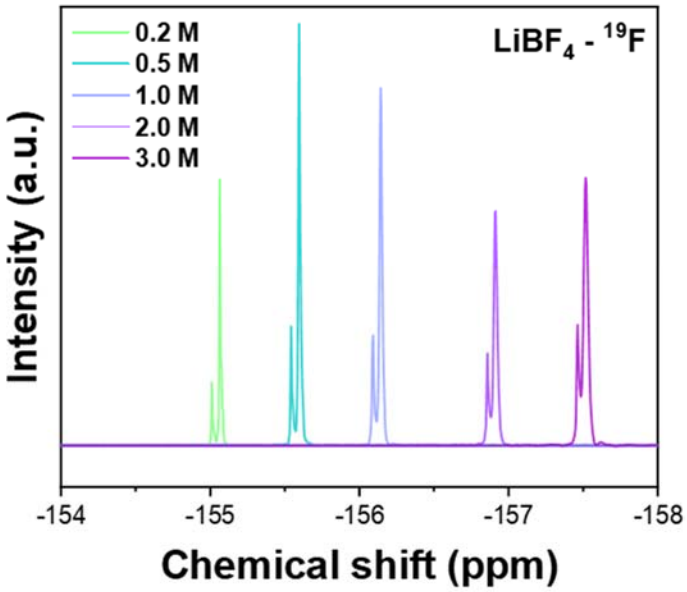


Figure S9. $^{19}F$ NMR spectra of different $LiBF_4$ electrolyte concentrations.

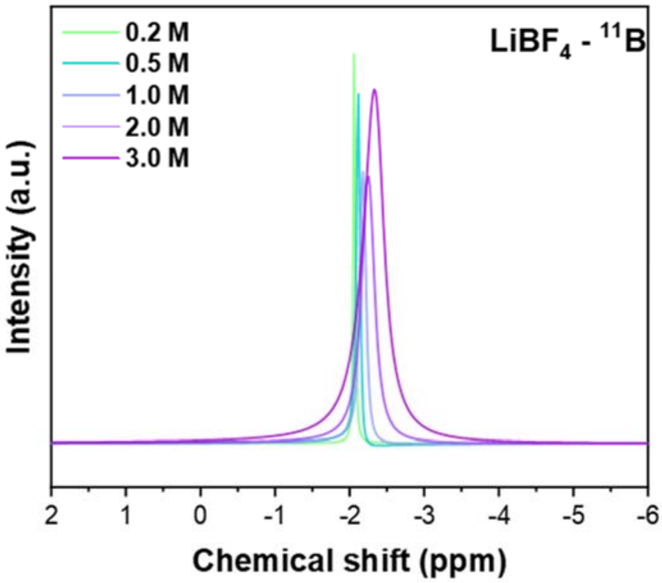


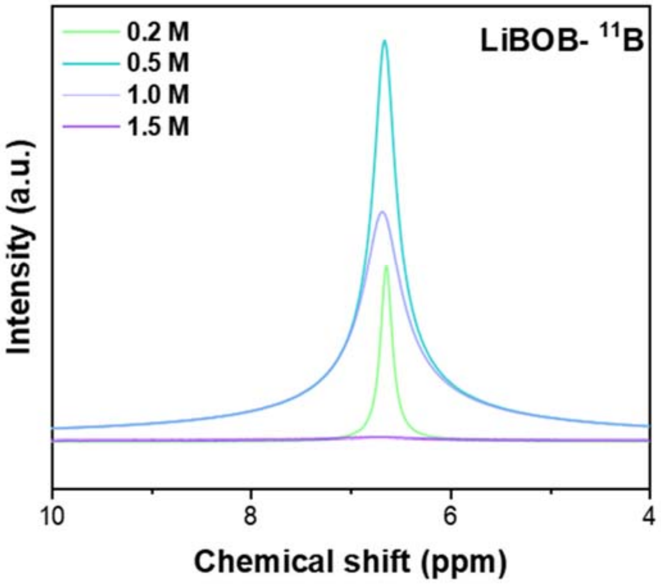


Figure S10. $^{11}B$ NMR spectra of different electrolyte concentrations of $LiBF_4$ and LiBOB.

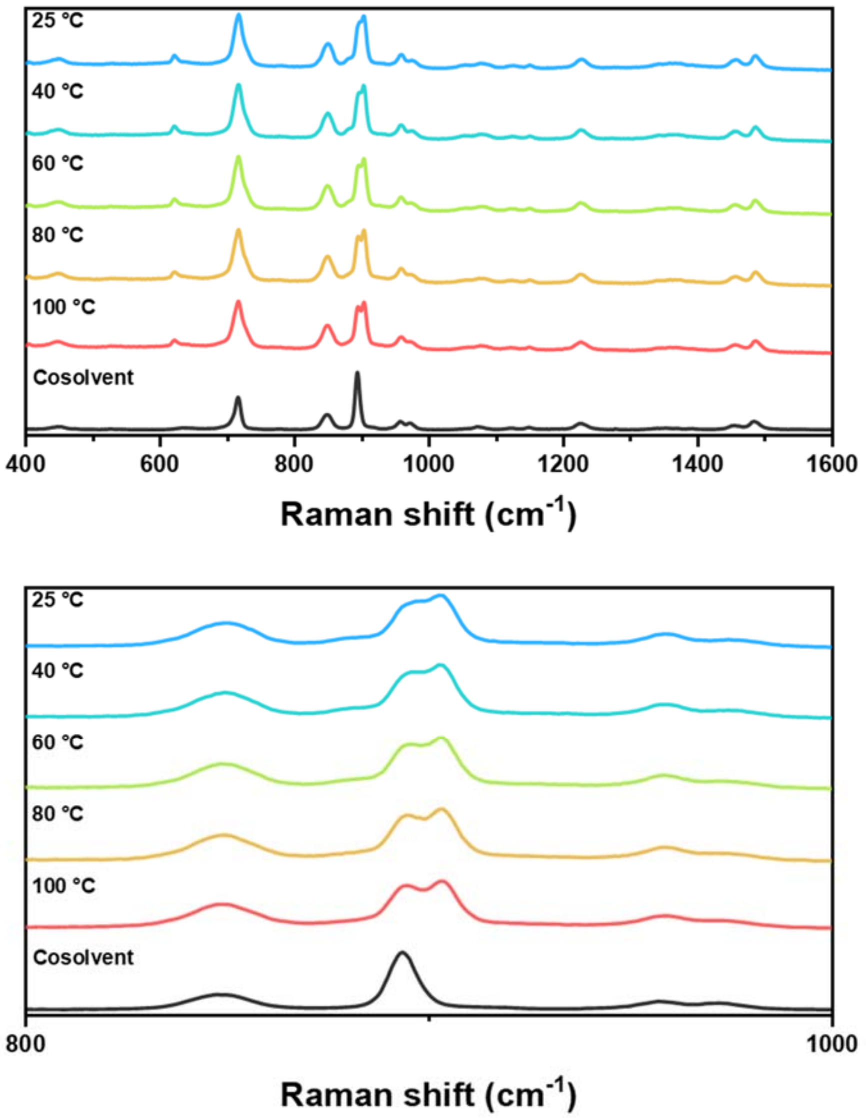


Figure S11. Raman spectra of 3.0 M electrolyte and cosolvent at different temperatures.

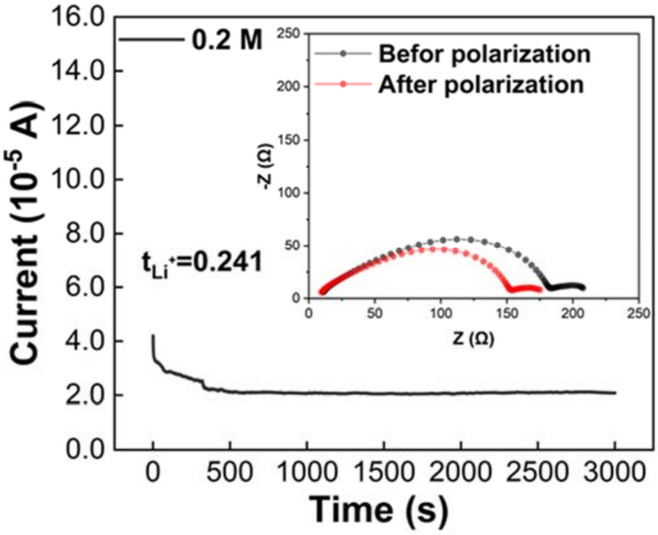


Figure S12. Polarization curve for $Li^+$ transference number of 0.2 M LiODFB.

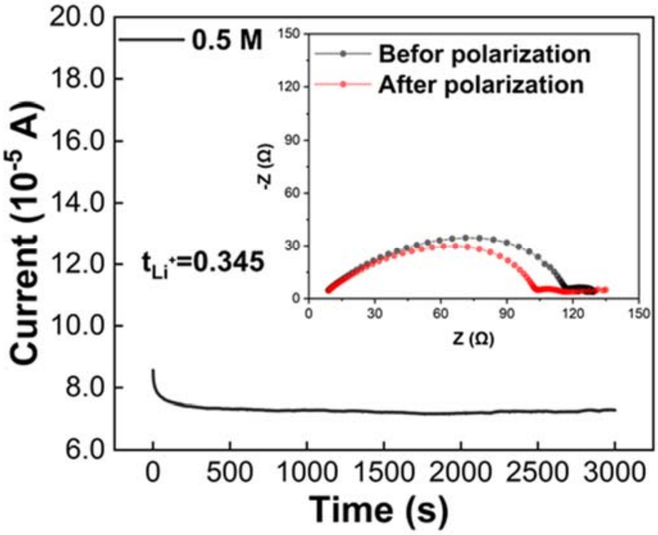


Figure S13. Polarization curve for $Li^+$ transference number of 0.5 M LiODFB.

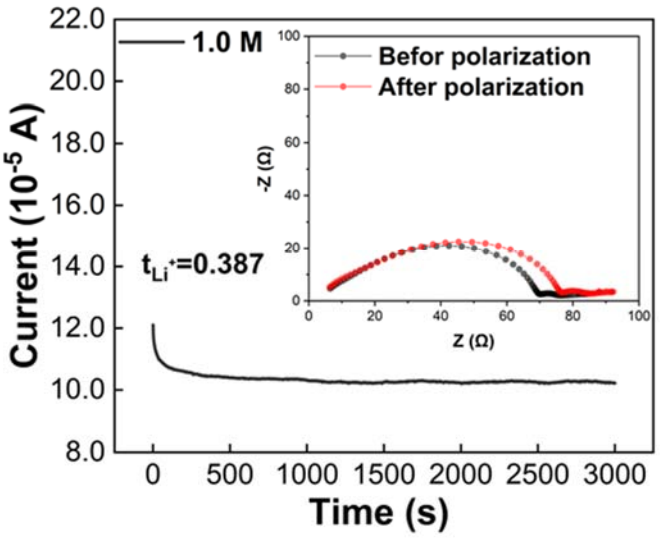


Figure S14. Polarization curve for $Li^+$ transference number of 1.0 M LiODFB.

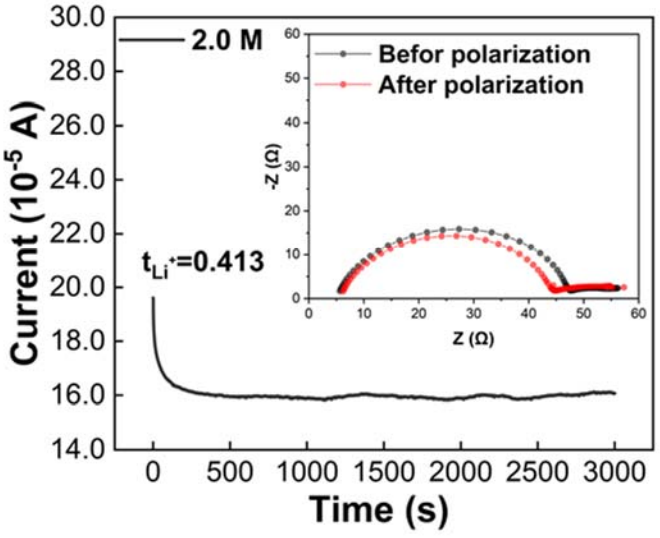


Figure S15. Polarization curve for $Li^+$ transference number of 2.0 M LiODFB.

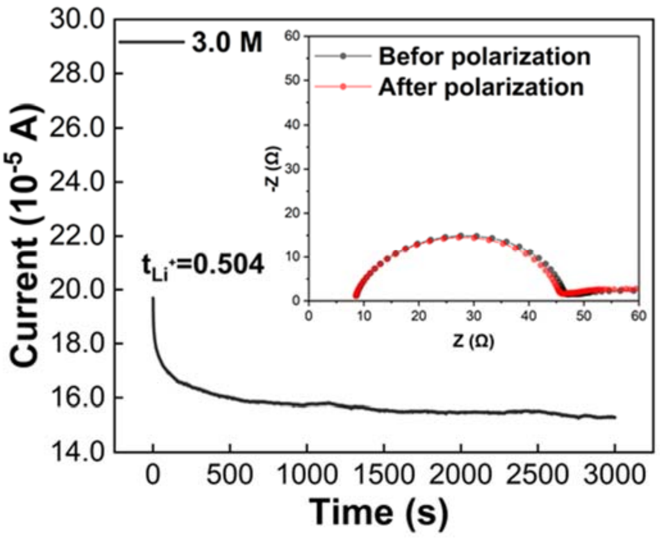


Figure S16. Polarization curve for $Li^+$ transference number of 3.0 M LiODFB.

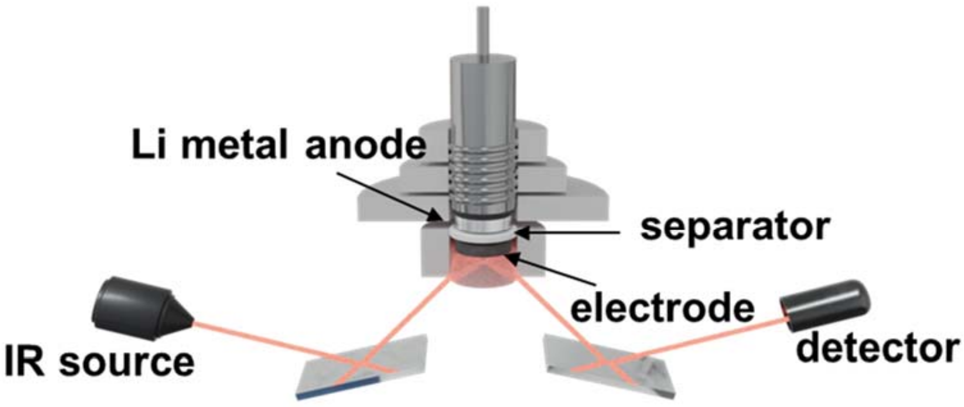


Figure S17. Schematic diagram of the electrochemical cell for in-situ FTIR measurements.

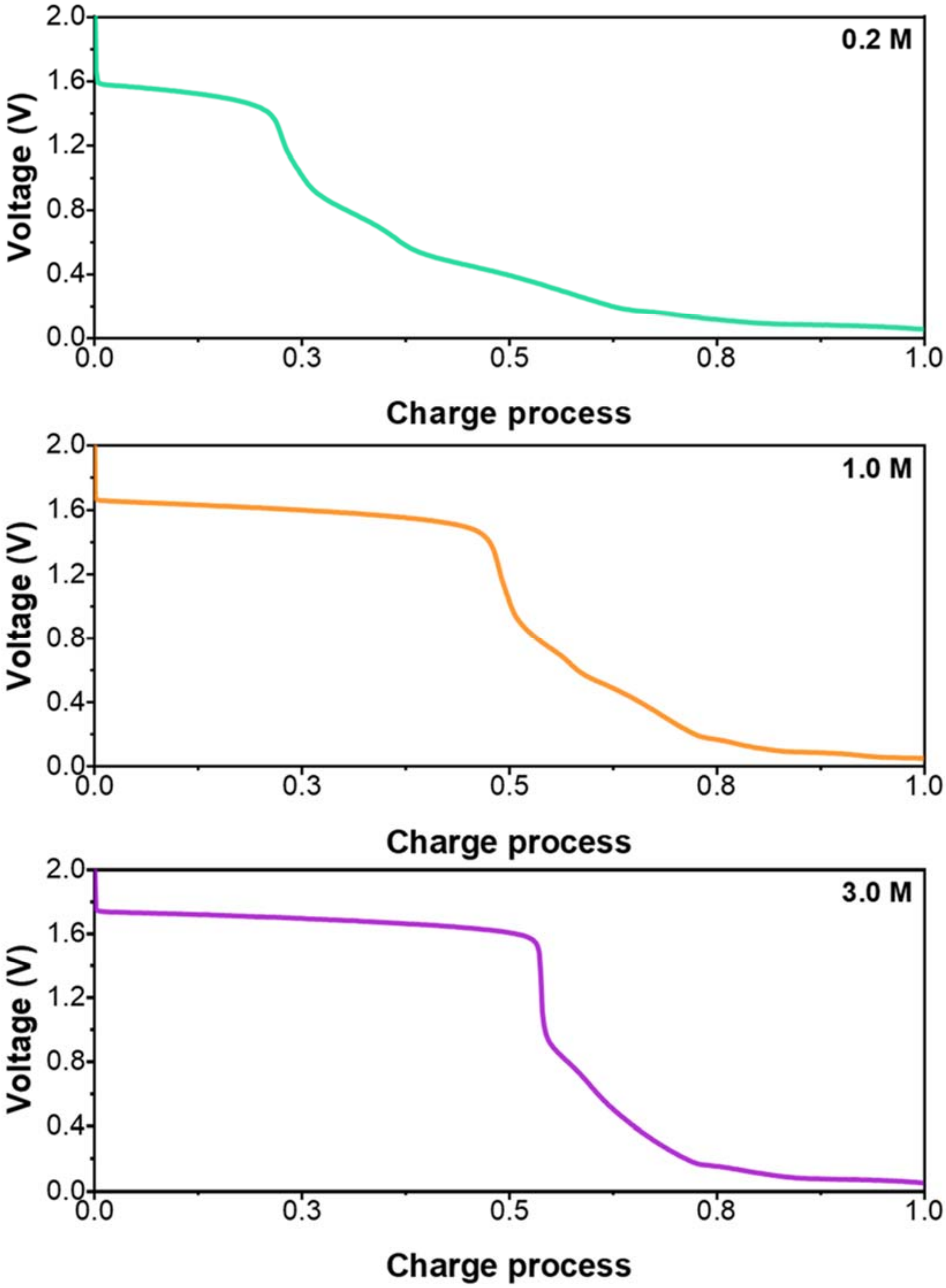


Figure S18. First discharge curve of in-situ FTIR Li/MCMB cell with 0.2, 1.0, and 3.0 M electrolyte.

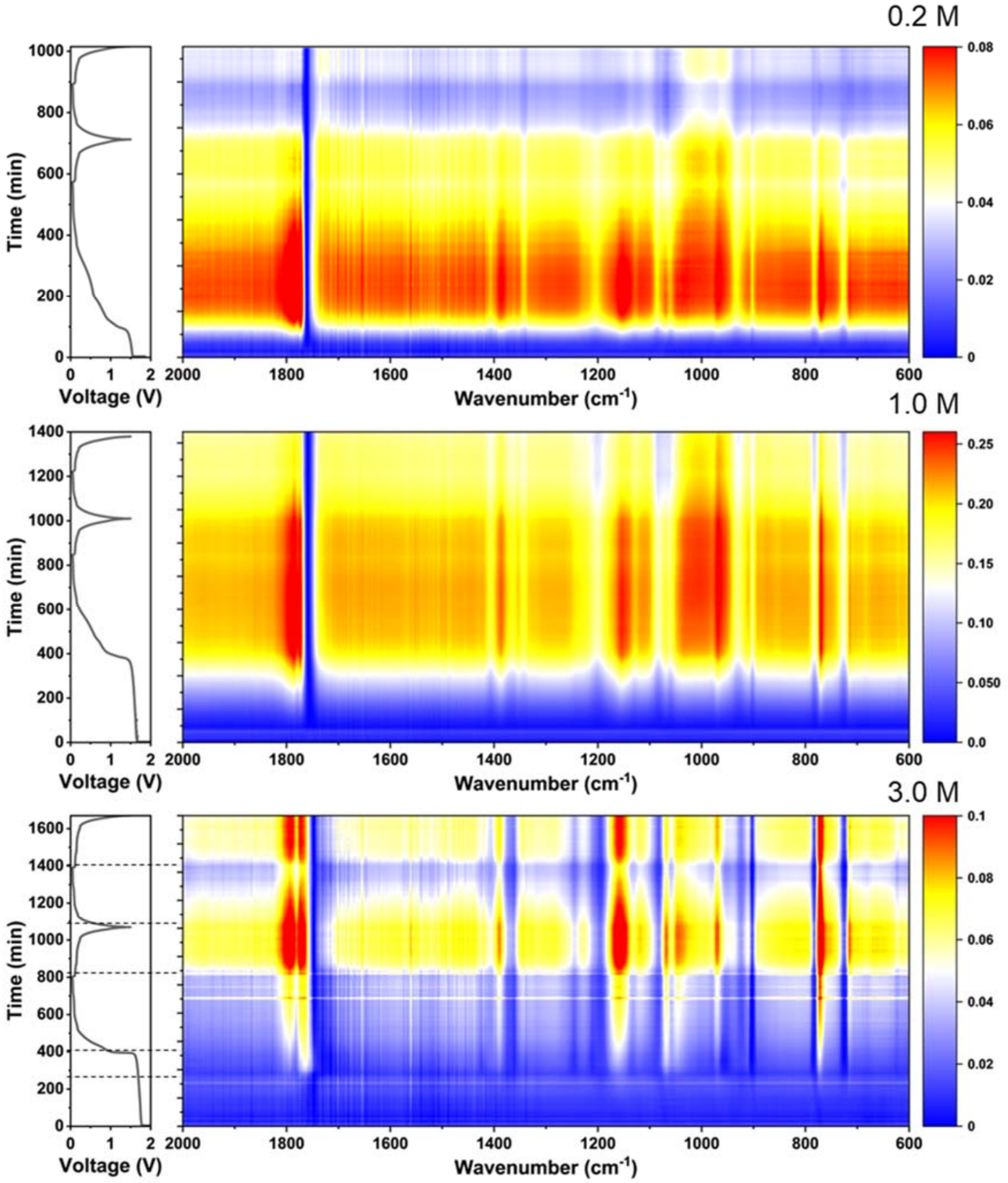


Figure S19. In-situ FTIR spectra of Li/MCMB cells with different electrolyte concentrations after baseline correction.

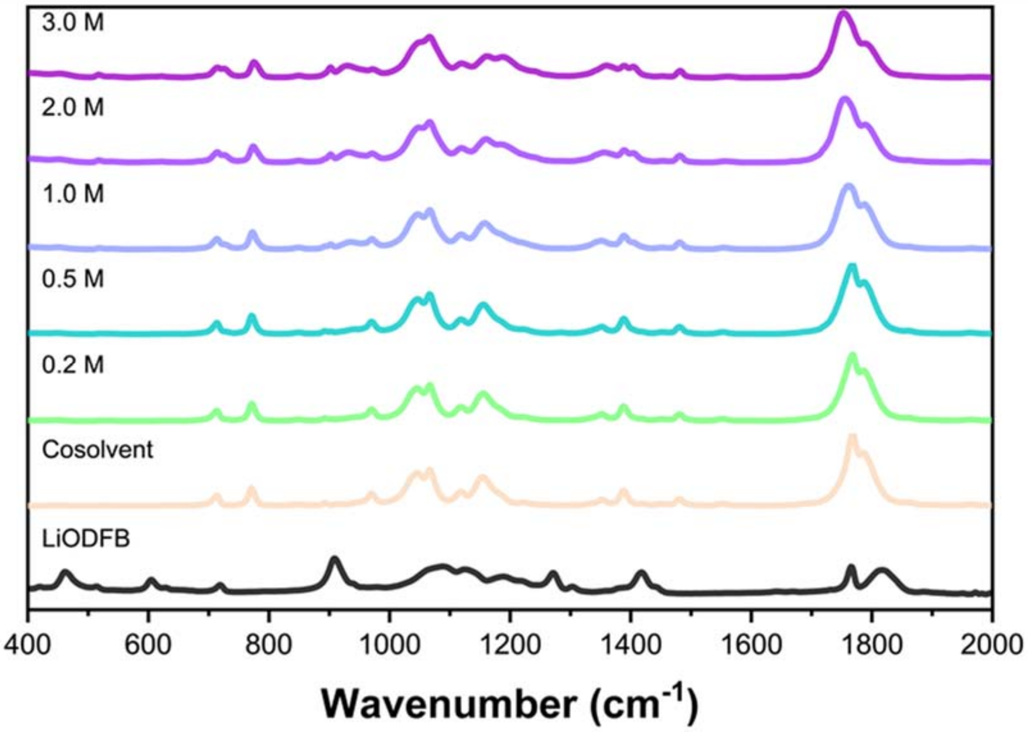


Figure S20. FTIR spectra of different electrolyte concentrations and pure LiODFB powder at the range of 400 – 2000 cm$^{-1}$.

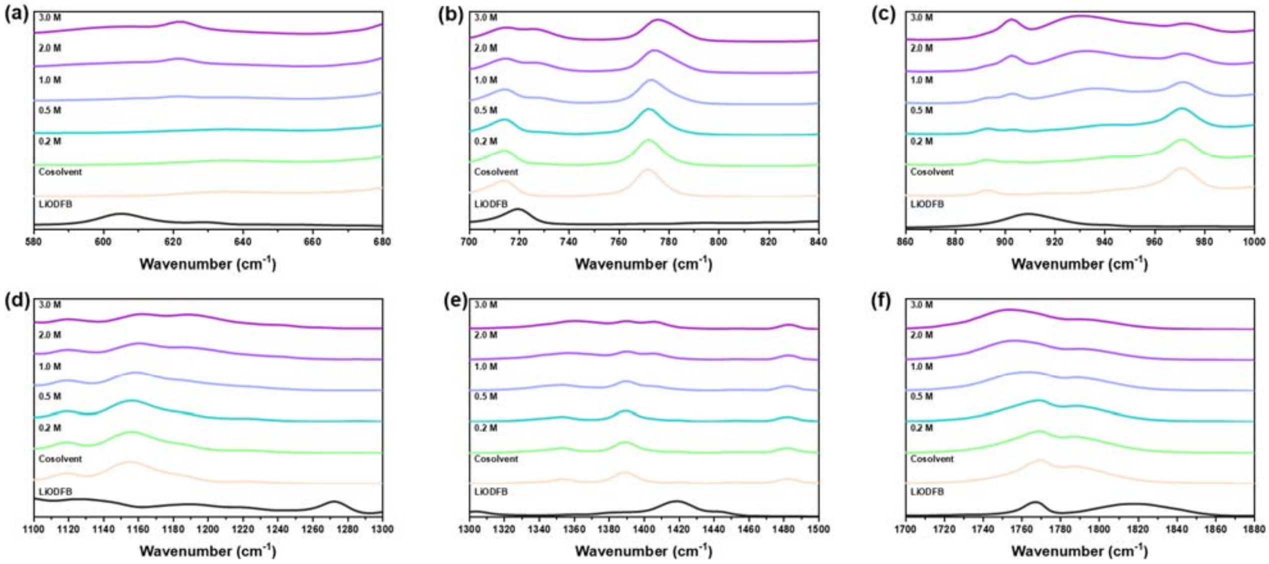


Figure S21. FTIR spectra of different electrolyte concentrations and pure LiODFB powder at localized range.

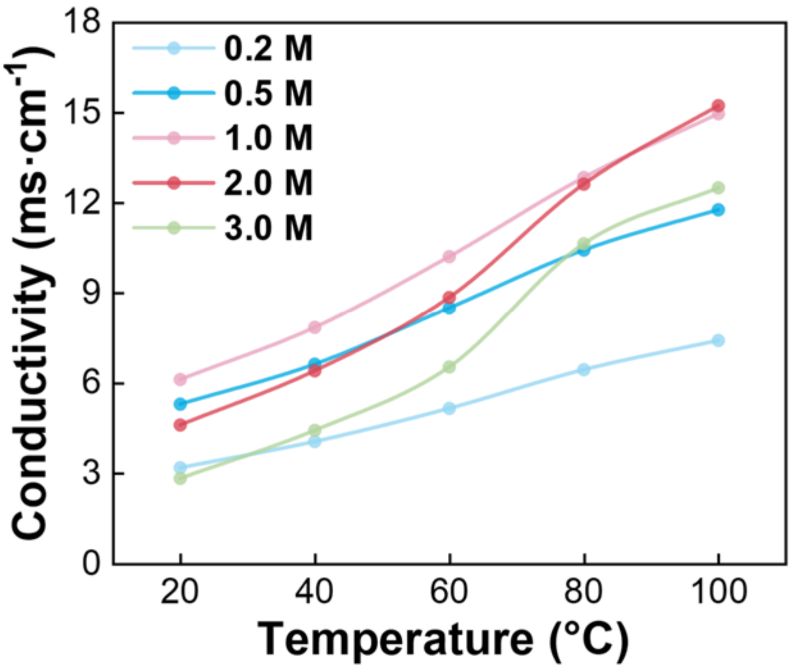


Figure S22. Conductivity test of different concentration electrolytes.

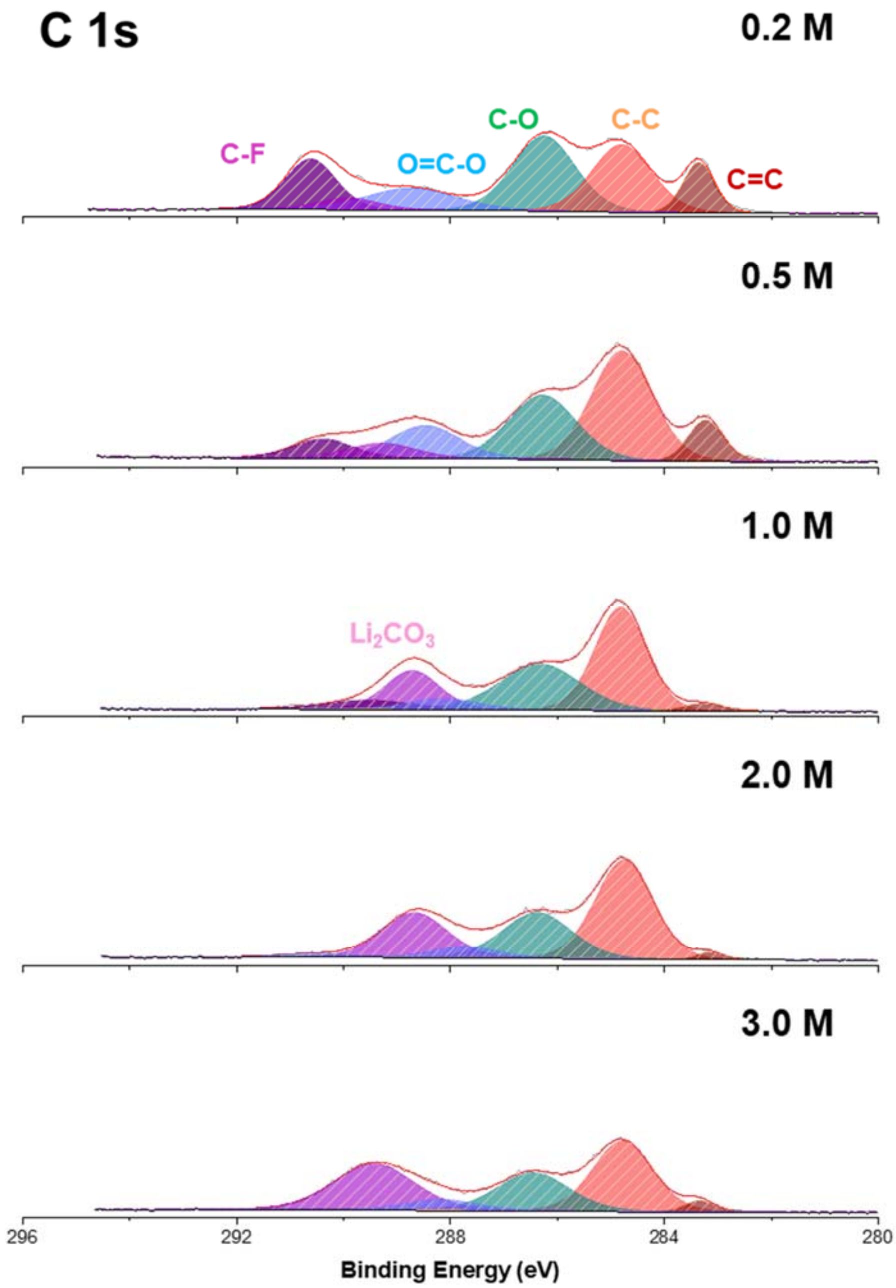


Figure S23. C 1s XPS spectra of the SEI layer.

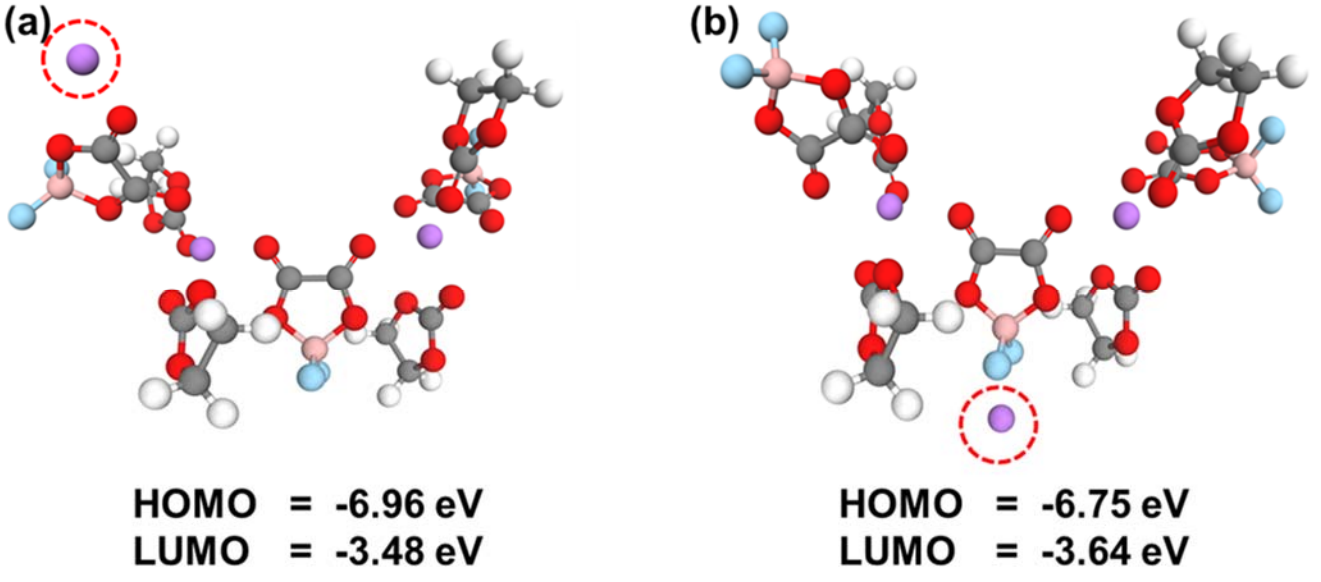


Figure S24. Typical solvation structure (a) C=O-$Li^+$ (b) C-F-$Li^+$ and their LUMO/HOMO.

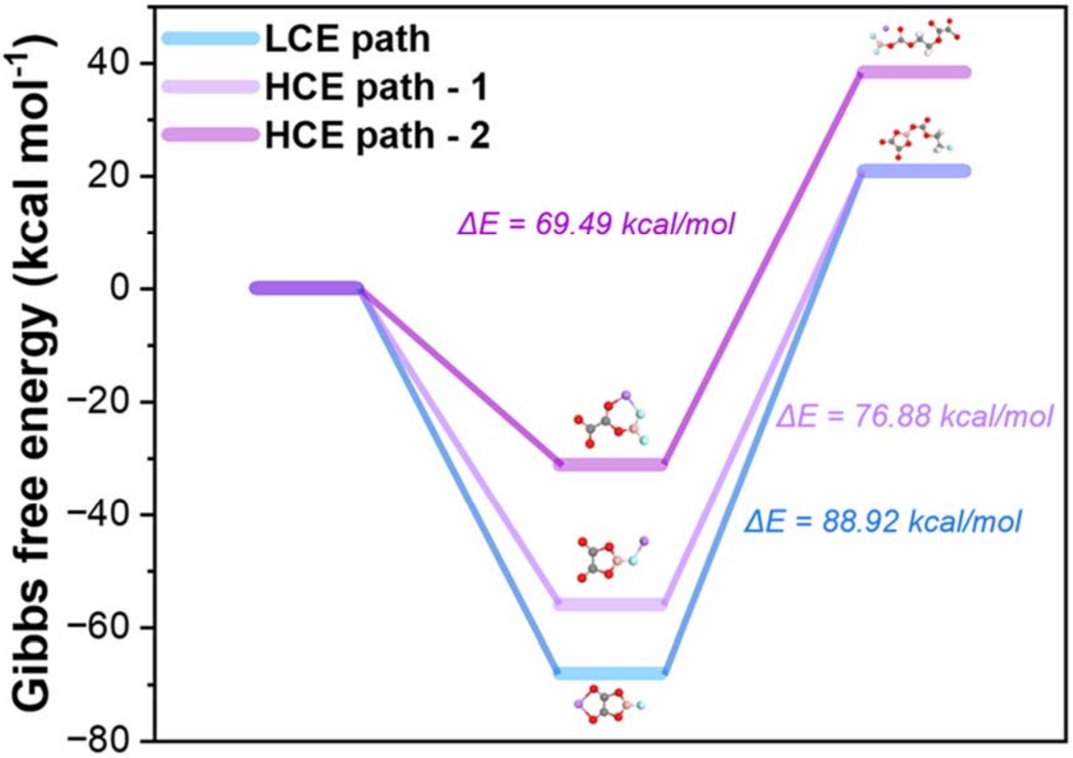


Figure S25. Typical solvation structure (a) C=O-$Li^+$ (b) C-F-$Li^+$

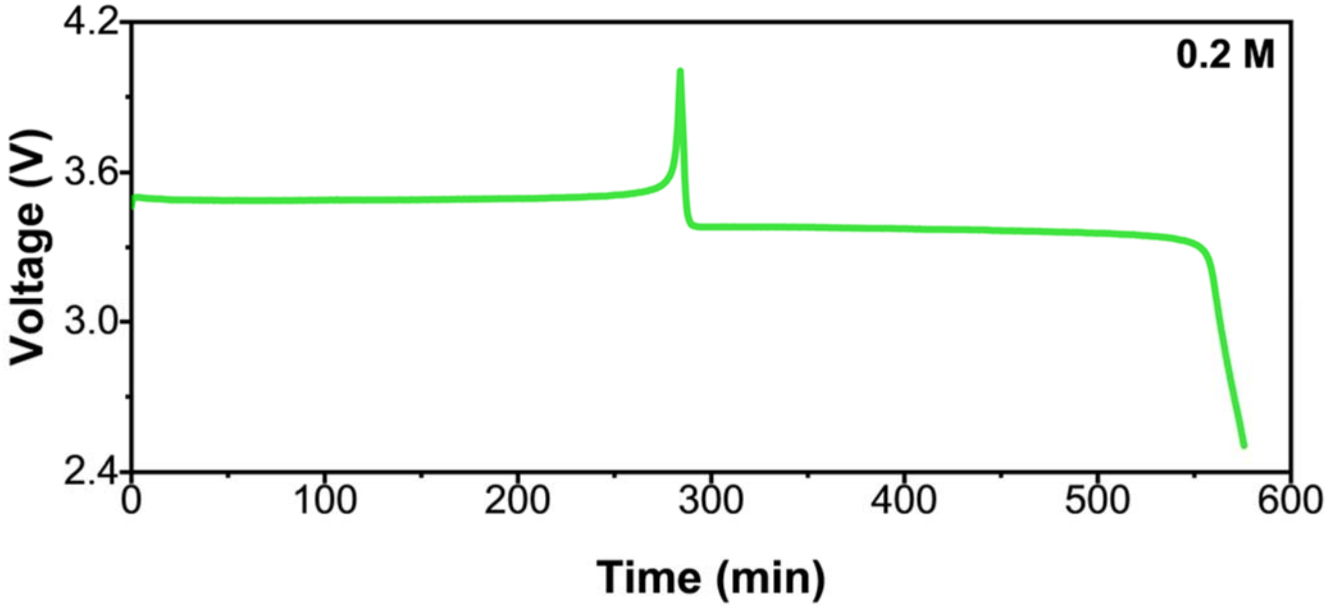


Figure S26. First cycle curve of in-situ FTIR Li/LFP cell with 0.2 M LiODFB electrolyte.

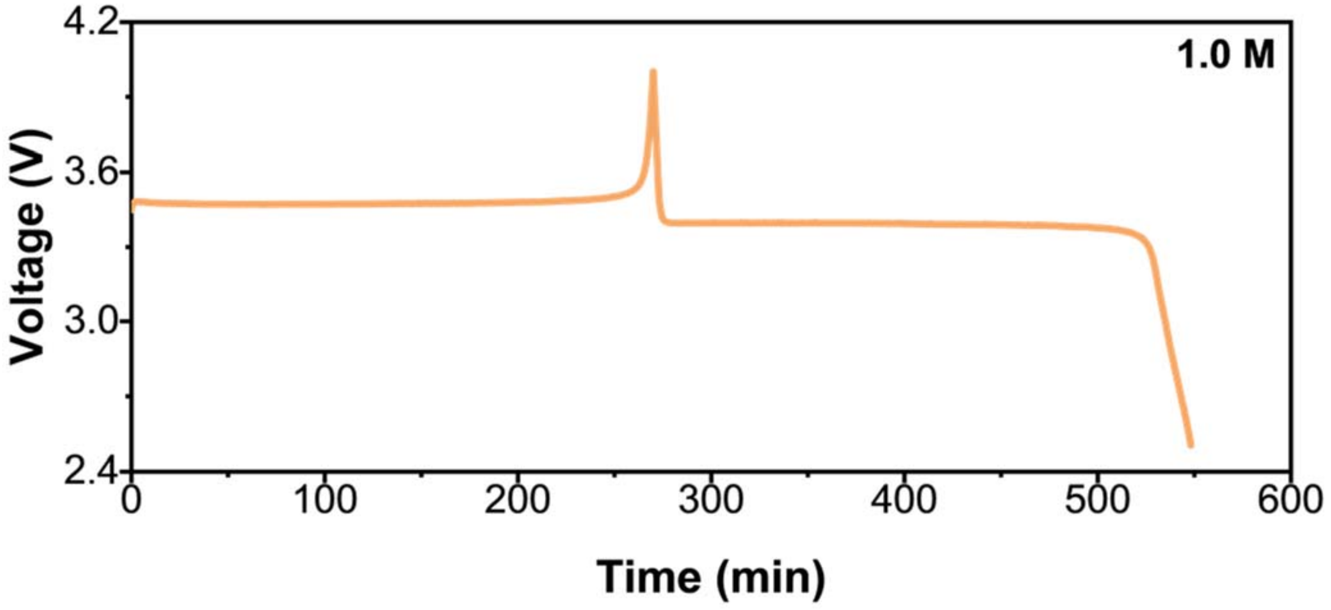


Figure S27. First cycle curve of in-situ FTIR Li/LFP cell with 1.0 M LiODFB electrolyte.

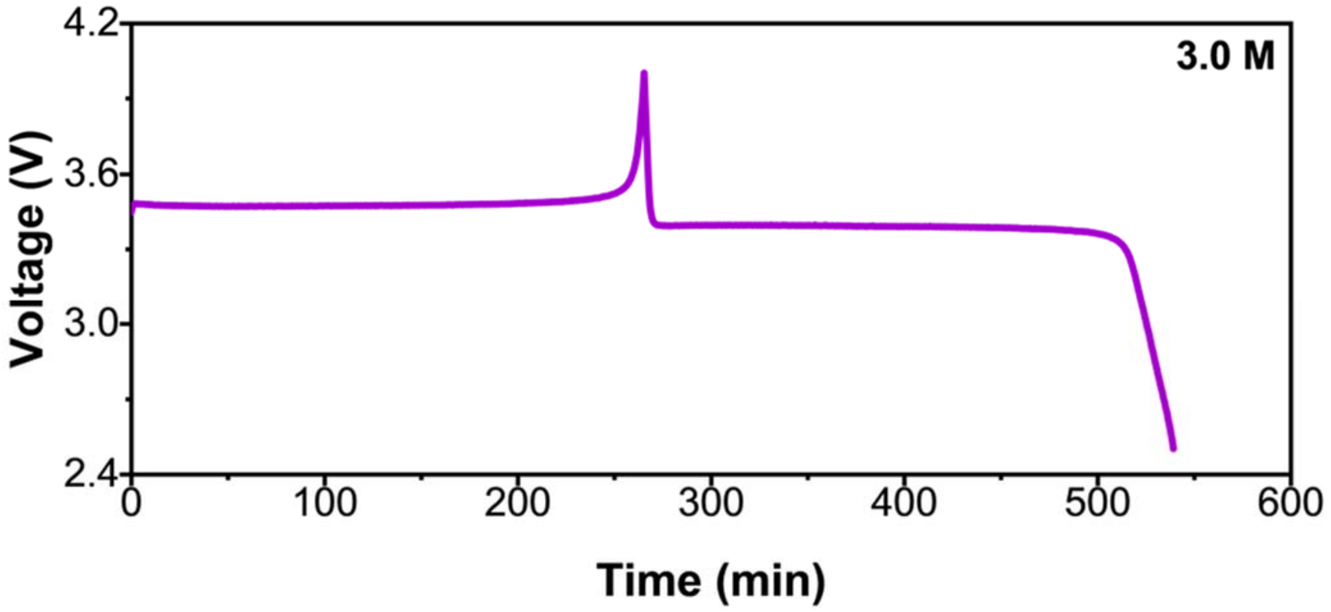


Figure S28. First cycle curve of in-situ FTIR Li/LFP cell with 3.0 M LiODFB electrolyte.

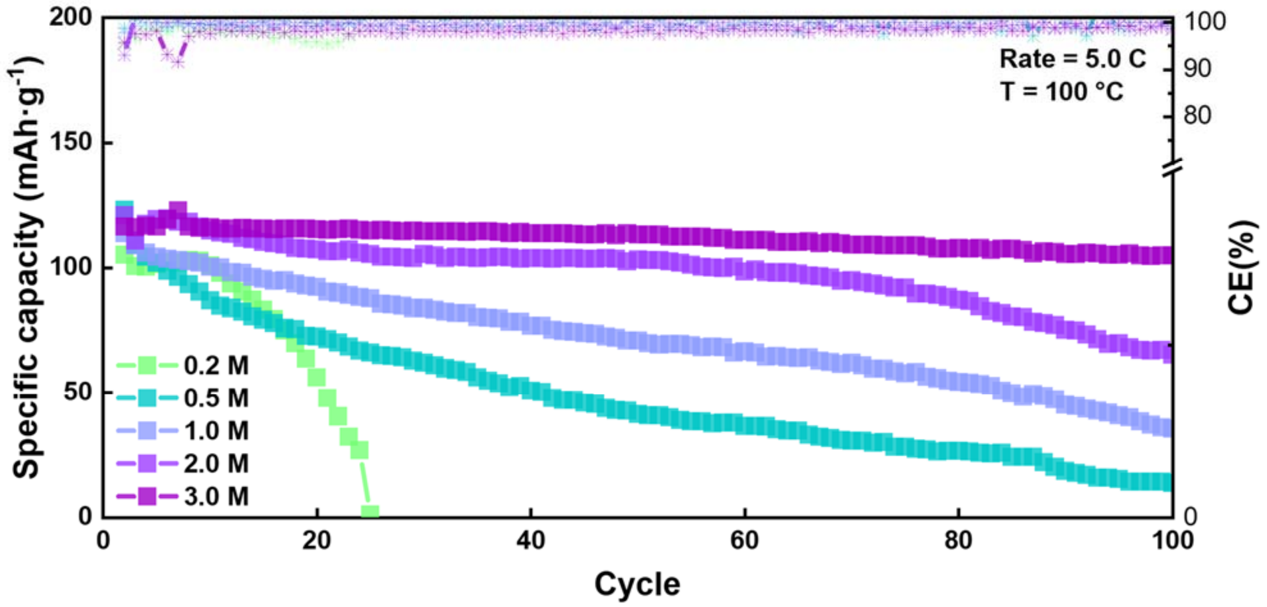


Figure S29. Long-term cycling performance of Li/LFP half-cells at a rate of 5.0 C at 100 °C.

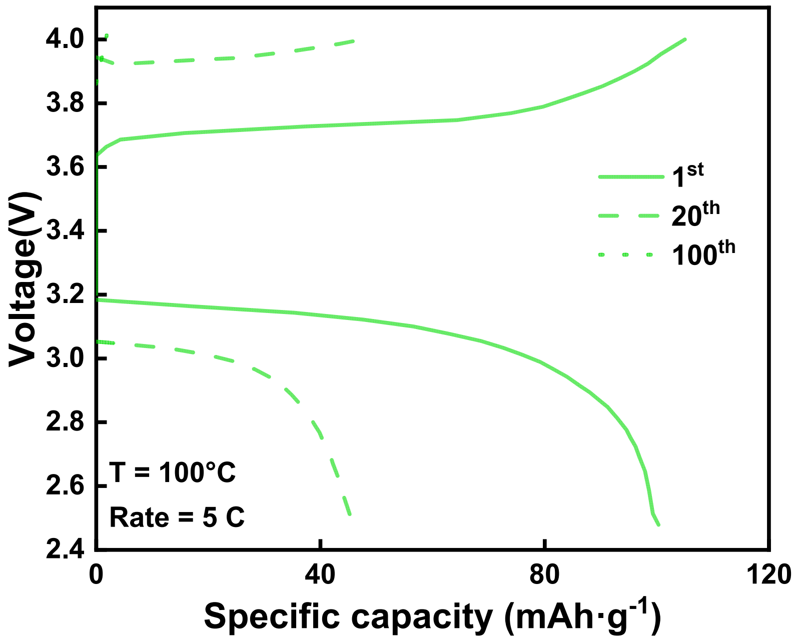


Figure S30. Discharge-charge curves of 1st, 20th, and 100th cycle of Li/LFP cell with 0.2 M LiODFB electrolyte after 5.0 C cycling at 100 °C.

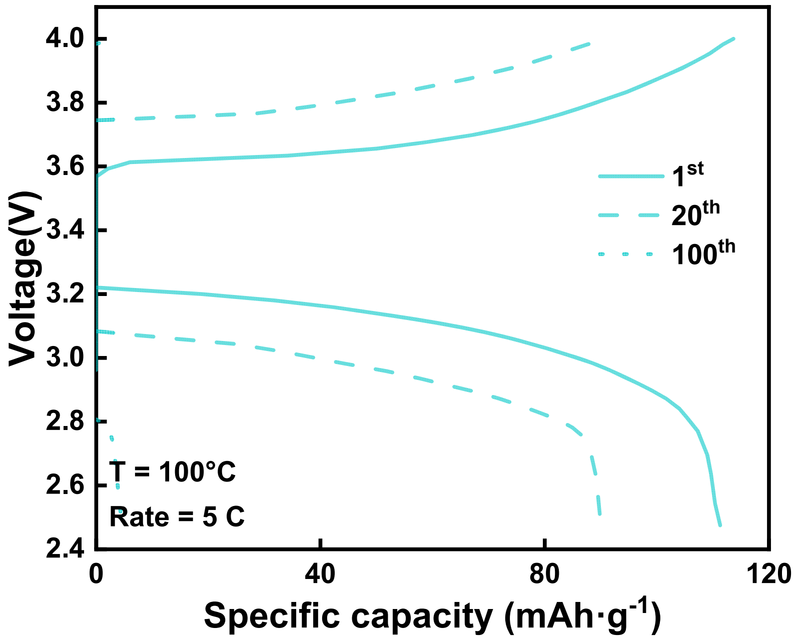


Figure S31. Discharge-charge curves of 1st, 20th, and 100th cycle of Li/LFP cell with 0.5 M LiODFB electrolyte after 5.0 C cycling at 100 °C.

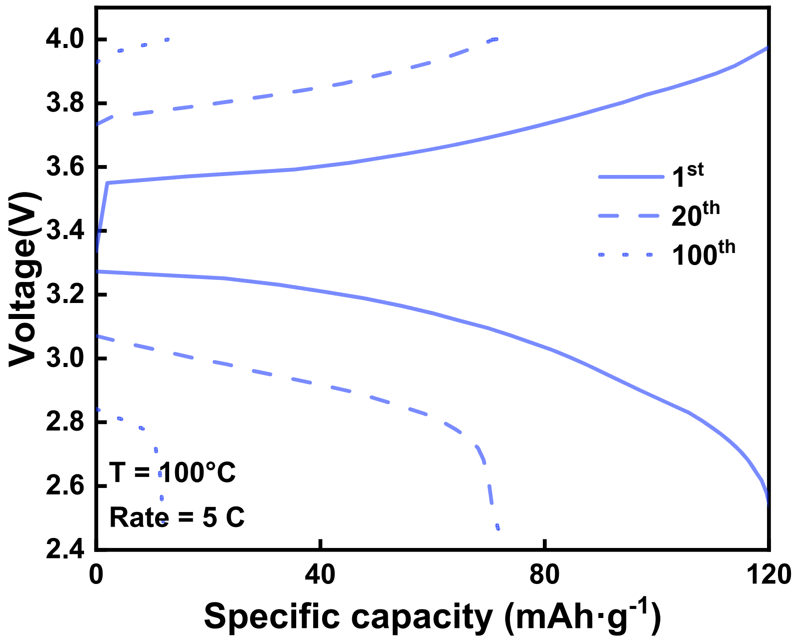


Figure S32. Discharge-charge curves of 1st, 20th, and 100th cycle of Li/LFP cell with 1.0 M LiODFB electrolyte after 5.0 C cycling at 100 °C.

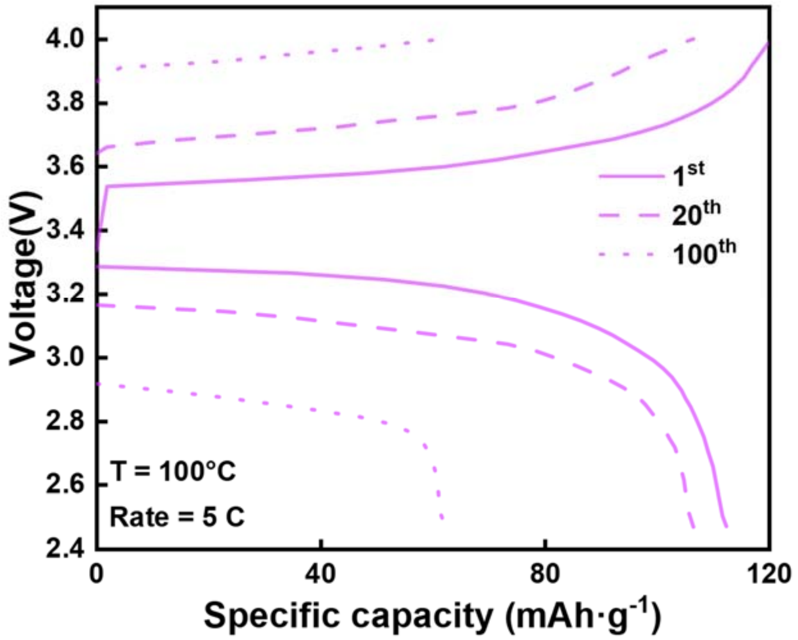


Figure S33. Discharge-charge curves of 1st, 20th, and 100th cycle of Li/LFP cell with 2.0 M LiODFB electrolyte after 5.0 C cycling at 100 °C.

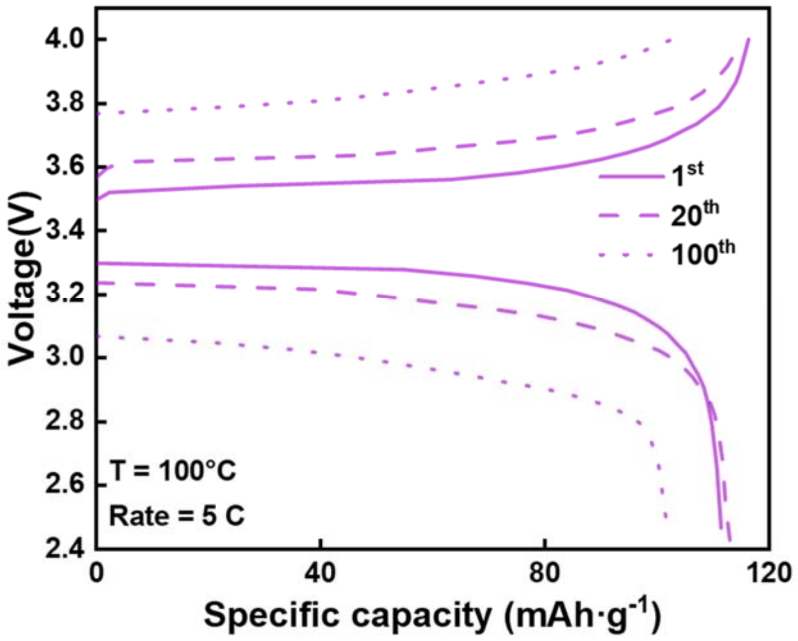


Figure S34.Discharge-charge curves of 1st, 20th, and 100th cycle of Li/LFP cell with 3.0 M LiODFB electrolyte after 5.0 C cycling at 100 °C.

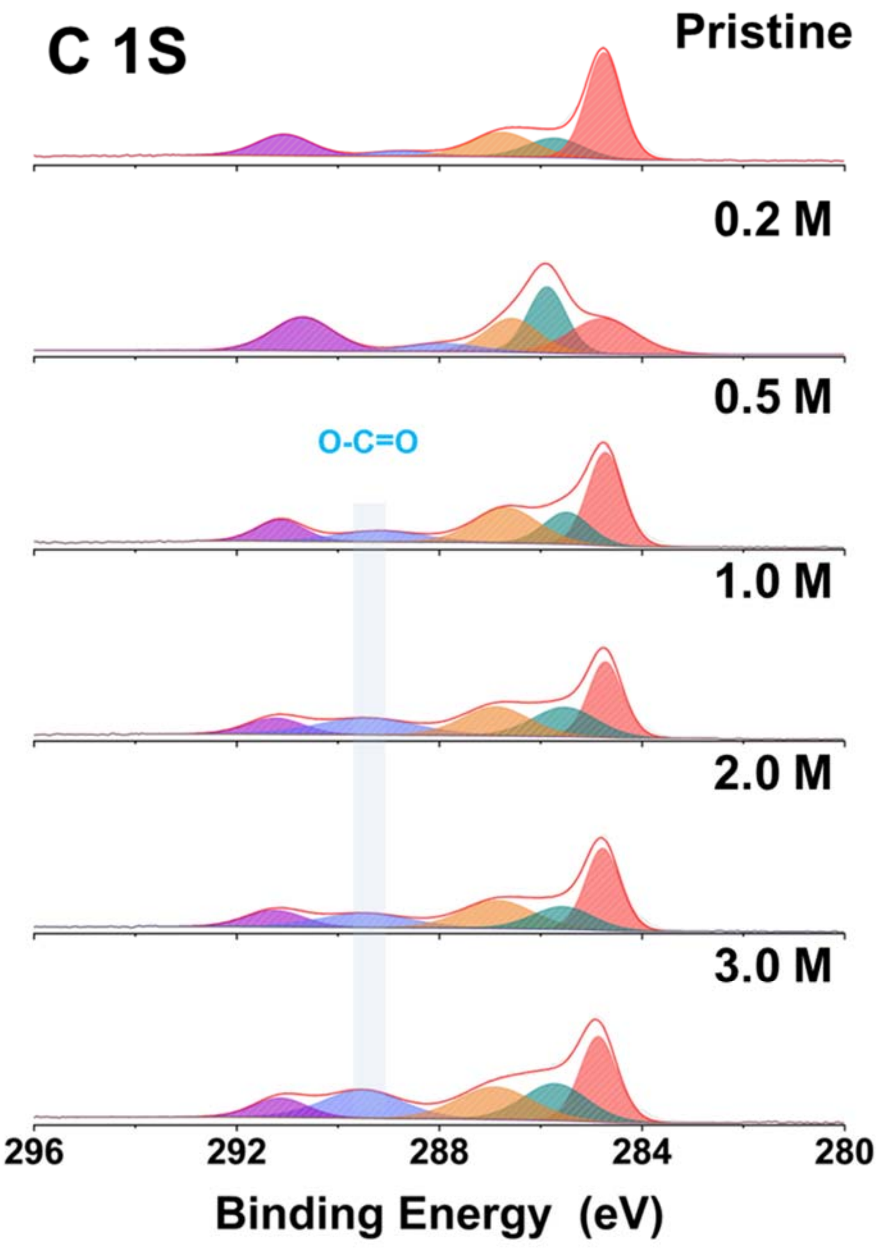


Figure S35. C 1s XPS spectra of the CEI layer.

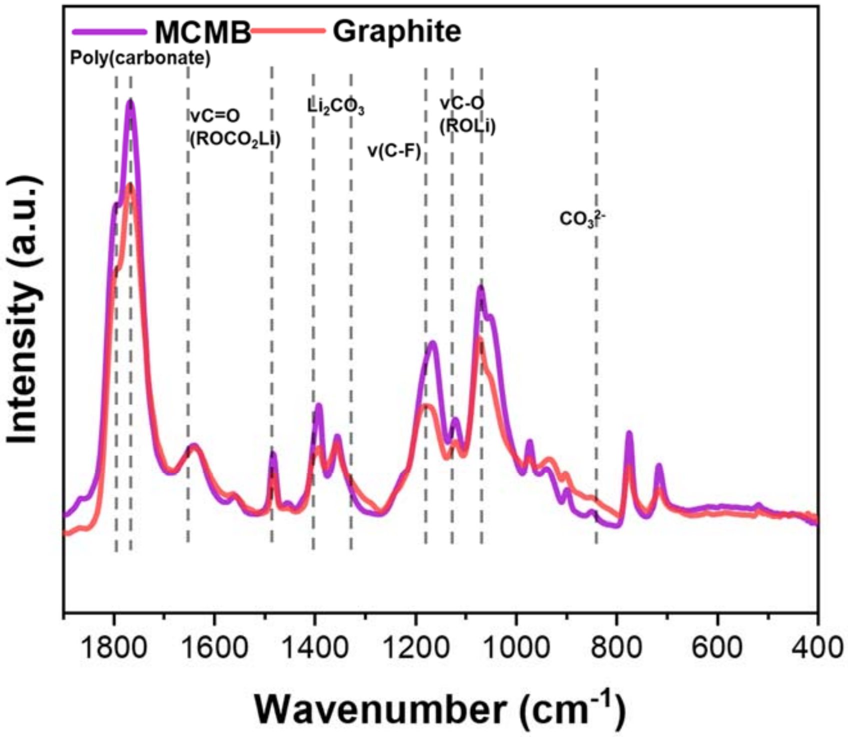


Figure S36. FTIR spectra of cycled MCMB and graphite anodes in 3.0 M concentration electrolytes at 100 °C.

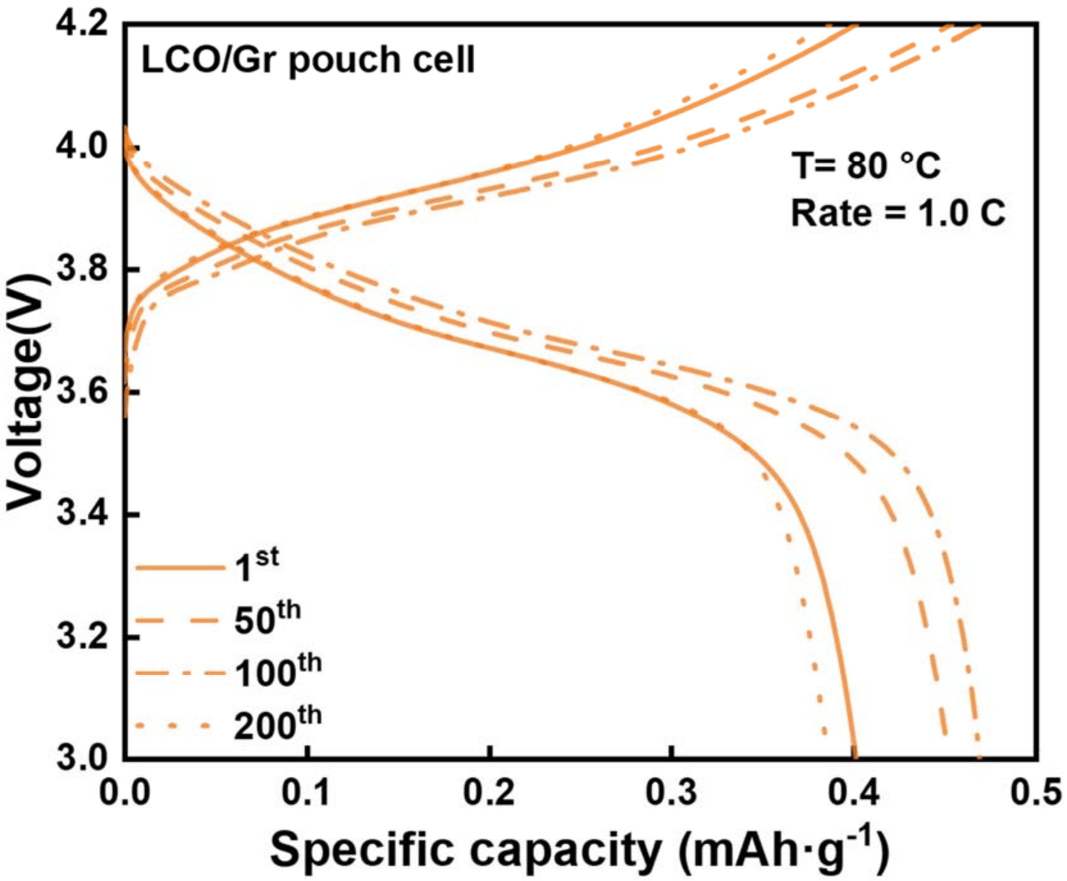


Figure S37.Discharge-charge curves of 1st, 50th,100th, and 200th cycle of LCO/Gr pouch cell with 3.0 M LiODFB electrolyte after 1.0 C cycling at 80 °C.

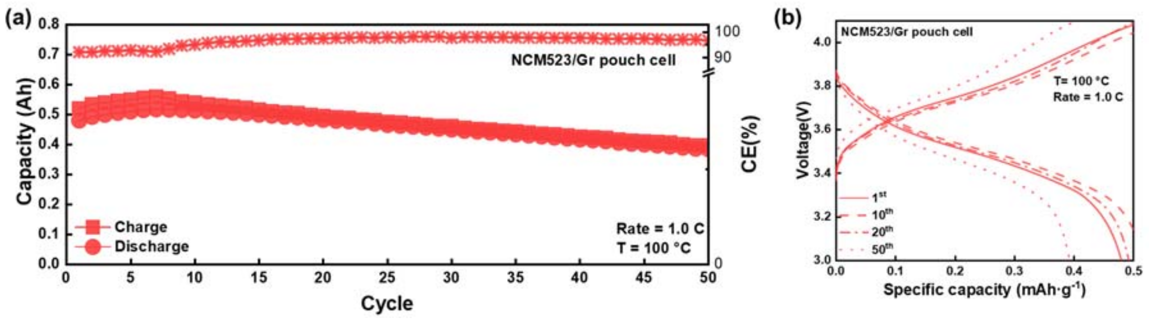


Figure S38. (a) Long-term performance of the NCM523/Gr pouch cell at 100 °C; (b) discharge-charge curves of 1st, 50th, 100th, and 200th cycle of NCM523/Gr pouch cell with 3.0 M LiODFB electrolyte after 1.0 C cycling at 100 °C.

**Supporting Tables**

| Electrolyte | Cell configuration | Cycle number (Temperature) | Capacity retention (%) | Ref |
|---|---|---|---|---|
| LiODFB-PC/EC | LCO/Gr (pouch cells) | 200 (80 °C) | 82.4 | This work |
| | LFP/Li (coin cells) | 200 (100 °C) | 90.3 | |
| | NCM523/Gr (pouch cells) | 50 (100 ° C) | 80.0 | |
| LiODFB-EC/DEC+1% PTSE | LCO/Gr (pouch cells) | 100 (80 °C) | 92.3 | [9] |
| LiFSI/LiODFB-FEC/TEGDNE | LFP/Gr (pouch cells) | 200 (70 °C) | 71.0 | [10] |
| LiODFB/LiFSI-DMS | LCO/Gr (coin cells) | 100 (60 °C) | 80.0 | [11] |
| LiBOB-TEP/MFE+2%VC | NCM811/Gr (coin cells) | 300 (55 °C) | 75.0 | [12] |
| $LiPF_6$-MDFA/PFPN/FEC | NCM811/Gr (coin cells) | 100 (60 °C) | 77.7 | [13] |
| LiODFB-EC/DMC | LCO/Gr (coin cells) | 160 (90 °C) | 97.0 | [14] |

Table S1. Comparison of different full cells in high temperatures, capacity retention (%) after more than 100 cycles, and maximum cycle numbers with previous studies.

**Reference**

[1] a) J. Alvarado, M. A. Schroeder, T. P. Pollard, X. F. Wang, J. Z. Lee, M. H. Zhang, T. Wynn, M. Ding, O. Borodin, Y. S. Meng, K. Xu, Energy & Environmental Science **2019**, 12, 780; b) Y. Yang, Y. Yin, D. M. Davies, M. Zhang, M. Mayer, Y. Zhang, E. S. Sablina, S. Wang, J. Z. Lee, O. Borodin, C. S. Rustomji, Y. S. Meng, Energy & Environmental Science **2020**, 13, 2209.

[2] M. J. Abraham, T. Murtola, R. Schulz, S. Páll, J. C. Smith, B. Hess, E. Lindahl, SoftwareX **2015**, 1-2, 19.

[3] W. Humphrey, A. Dalke, K. Schulten, Journal of Molecular Graphics **1996**, 14, 33.

[4] L. Martinez, R. Andrade, E. G. Birgin, J. M. Martinez, J Comput Chem **2009**, 30, 2157.

[5] a) J. Wang, R. M. Wolf, J. W. Caldwell, P. A. Kollman, D. A. Case, Journal of Computational Chemistry **2004**, 25, 1157; b) P. Han, W. Nie, G. Zhao, P. Gao, Journal of Molecular Liquids **2022**, 366.

[6] a) T. Lu, Sobtop, accessed: 2023; b) T. Lu, F. Chen, J Comput Chem **2012**, 33, 580; c) M. Schauperl, P. S. Nerenberg, H. Jang, L. P. Wang, C. I. Bayly, D. L. Mobley, M. K. Gilson, Commun Chem **2020**, 3.

[7] P. Li, B. P. Roberts, D. K. Chakravorty, K. M. Merz, Jr., J Chem Theory Comput **2013**, 9, 2733.

[8] B. Hess, H. Bekker, H. J. C. Berendsen, J. G. E. M. Fraaije, Journal of Computational Chemistry **1997**, 18, 1463.

[9] P. Xiao, H. Gao, Y. Chen, T. Teng, X. Yun, D. Lu, G. Zhou, Y. Zhao, B. Li, X. Zhou, C. Zheng, Angewandte Chemie International Edition **2024**, n/a, e202410982.
[10] L. Chen, J. Lu, Y. Wang, P. He, S. Huang, Y. Liu, Y. Wu, G. Cao, L. Wang, X. He, J. Qiu, H. Zhang, Energy Storage Materials **2022**, 49, 493.
[11] Y. Zhao, Z. Hu, Z. Zhao, X. Chen, S. Zhang, J. Gao, J. Luo, Journal of the American Chemical Society **2023**, 145, 22184.
[12] C. Yang, M. Zheng, R. Qu, H. Zhang, L. Yin, W. Hu, J. Han, J. Lu, Y. You, Advanced Materials **2024**, 36, 2307220.
[13] Y. Zou, Z. Ma, G. Liu, Q. Li, D. Yin, X. Shi, Z. Cao, Z. Tian, H. Kim, Y. Guo, C. Sun, L. Cavallo, L. Wang, H. N. Alshareef, Y.-K. Sun, J. Ming, Angewandte Chemie International Edition **2023**, 62, e202216189.
[14] M. Zhang, J. Zhang, J. Yang, J. Yao, Z. Chen, C. Lu, X. Du, Z. Zhang, H. Zhang, G. Cui, Chemical Communications **2019**, 55, 9785.
